# Synthesis of rare-earth metal and rare-earth metal-fluoride nanoparticles in ionic liquids and propylene carbonate

**DOI:** 10.3762/bjnano.9.180

**Published:** 2018-06-28

**Authors:** Marvin Siebels, Lukas Mai, Laura Schmolke, Kai Schütte, Juri Barthel, Junpei Yue, Jörg Thomas, Bernd M Smarsly, Anjana Devi, Roland A Fischer, Christoph Janiak

**Affiliations:** 1 Institut für Anorganische Chemie und Strukturchemie, Heinrich-Heine-Universität Düsseldorf, 40204 Düsseldorf, Germany. Fax: +49-211-81-12287; Tel: +49-211-81-12286; 2 Inorganic Materials Chemistry, Ruhr-Universität Bochum, 44801 Bochum, Germany; 3 Gemeinschaftslabor für Elektronenmikroskopie RWTH-Aachen, Ernst Ruska-Centrum für Mikroskopie und Spektroskopie mit Elektronen, 52425 Jülich, Germany; 4 Physikalisch-Chemisches Institut, Justus-Liebig-Universität Gießen, 35392 Gießen, Germany; 5 Department Structure and Nano-/Micromechanics of Materials, Max-Planck-Institut für Eisenforschung GmbH, 40237 Düsseldorf, Germany; 6 Department of Chemistry, Technische Universität München, 85748 Garching, Germany

**Keywords:** ionic liquids, metal amidinates, rare-earth metal-fluoride nanoparticles, rare-earth metal nanoparticles, soft wet-chemical synthesis

## Abstract

Decomposition of rare-earth tris(*N*,*N*′-diisopropyl-2-methylamidinato)metal(III) complexes [RE{MeC(N(iPr)_2_)}_3_] (RE(amd)_3_; RE = Pr(III), Gd(III), Er(III)) and tris(2,2,6,6-tetramethyl-3,5-heptanedionato)europium(III) (Eu(dpm)_3_) induced by microwave heating in the ionic liquids (ILs) 1-butyl-3-methylimidazolium tetrafluoroborate ([BMIm][BF_4_]), 1-butyl-3-methylimidazolium bis(trifluoromethylsulfonyl)imide ([BMIm][NTf_2_]) and in propylene carbonate (PC) yield oxide-free rare-earth metal nanoparticles (RE-NPs) in [BMIm][NTf_2_] and PC for RE = Pr, Gd and Er or rare-earth metal-fluoride nanoparticles (REF_3_-NPs) in the fluoride-donating IL [BMIm][BF_4_] for RE = Pr, Eu, Gd and Er. The crystalline phases and the absence of significant oxide impurities in RE-NPs and REF_3_-NPs were verified by powder X-ray diffraction (PXRD), selected area electron diffraction (SAED) and high-resolution X-ray photoelectron spectroscopy (XPS). The size distributions of the nanoparticles were determined by transmission electron microscopy (TEM) and high-angle annular dark-field scanning transmission electron microscopy (HAADF-STEM) to an average diameter of (11 ± 6) to (38 ± 17) nm for the REF_3_-NPs from [BMIm][BF_4_]. The RE-NPs from [BMIm][NTf_2_] or PC showed diameters of (1.5 ± 0.5) to (5 ± 1) nm. The characterization was completed by energy-dispersive X-ray spectroscopy (EDX).

## Introduction

Rare-earth (RE) elements gain increasing importance in materials science and modern chemistry [1–3]. Special attention has been paid to nanoscaled rare-earth metal particles [4–6]. In addition to the oxido and nitrido compounds, the rare-earth fluorides have interesting photo physical and electrochemical properties. An important representative of this category are AREF_4_ compounds (A = alkali metal), with unique optical, magnetic and piezoelectric properties [7]. They are applied in solid-state lasers, three-dimensional flat-panel displays, and low-intensity IR imaging [8]. Syntheses of these AREF_4_-type compounds are based on the liquid precipitation reaction between soluble rare-earth metal salts and alkaline fluorides. A co-thermolysis of Na(CF_3_COO) and RE(CF_3_COO) in oleic acid/oleylamine for the synthesis of NaREF_4_ (RE = Er(III), Tm(III)) has also been described [8]. One problem of these syntheses is that the obtained rare-earth fluoride particles were not phase-pure [8].

**Scheme 1:**
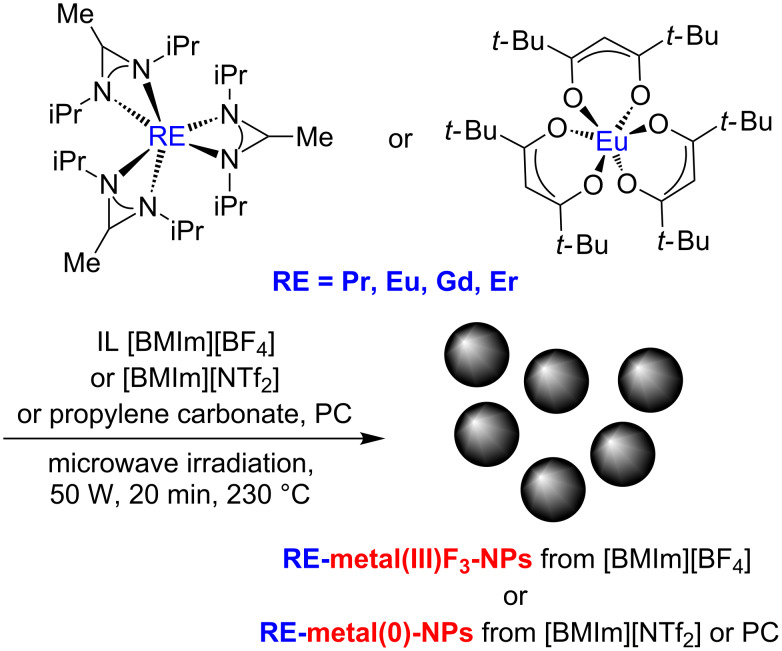
Synthesis of REF_3_-NPs or RE-NPs from the rare-earth metal amidinates RE(amd)_3_ and Eu(dpm)_3_ by microwave-assisted thermal decomposition in the ionic liquids [BMIm][BF_4_], [BMIm][NTf_2_] and PC.

An alternative method for synthesizing rare-earth metal-fluoride nanoparticles is the use of rare-earth metal amidinates as precursors [9–12]. Metal amidinates are coordination compounds [13–14] and used, for example, as catalysts in the polymerization of olefins [15–16] and as precursors in chemical vapor (CVD) processes of rare-earth materials such as oxides and nitrides [17–19]. It is especially advantageous that the decomposition products from the amidinate ligand are gaseous so that product contamination is minimized [20]. Herein, we report the use of rare-earth amidinates RE(amd)_3_ with RE = Pr(III), Gd(III), Er(III) and of tris(2,2,6,6-tetramethyl-3,5-heptanedionato)europium(III) (Eu(dpm)_3_) as precursors for rare-earth metal-fluoride nanoparticles (REF_3_-NPs) by microwave-assisted thermal syntheses in the ionic liquid (IL) 1-butyl-3-methylimidazolium tetrafluoroborate ([BMIm][BF_4_]) with the reactive BF_4_ anion as a fluoride source (Scheme 1). For the synthesis of EuF_3_-NPs, the precursor Eu(dpm)_3_ was used, since a europium amidinate, Eu(amd)_3_ is not yet known in the literature and therefore not available. In general metal-fluoride nanoparticles, are important in materials science and modern chemistry [21–22].

However, in the absence of fluoride donors we obtained oxide-free rare-earth metal nanoparticles (RE-NPs) of Pr, Gd and Er (Scheme 1). By using either the chemically more inert IL 1-butyl-3-methylimidazolium bis(trifluoromethylsulfonyl)imide ([BMIm][NTf_2_]) or propylene carbonate (PC) as reaction media RE-NPs were produced. This is an unusual and quite interesting result that we wish to highlight at this point. There exist only few reports on the wet-chemical synthesis of nanoparticles of rare-earth metals or rare-earth metal containing alloys or intermetallic phases. Solution synthesis of any oxide-free RE-NP was first reported by Wagner and co-workers using alkalide reduction of GdCl_3_ in THF solution [6]. Scalable air- and water-stable core–shell Gd@Au-NPs were obtained by using the same strategy [23]. Rare-earth metal containing intermetallic nano-phases have been suggested as novel materials for various catalytic applications [24]. For example, Pt_3_Y and Pt_5_Gd were predicted to be more active as Pt in the oxygen reduction reaction (ORR) [25]. Nevertheless, the high reduction potentials of rare-earth metal ions (typically below −2.0 V vs NHE) cause much difficulties regarding the chemical reduction of any chosen precursor and the prevention of post-synthesis oxidation or contamination of the RE-NPs. Recently, Alivisatos and co-authors reported on the synthesis of Pt_3_Y and other so-called early–late intermetallic nanoparticles by a solvent-free route employing a melt of the reducing agent (Na/K)BEt_3_H [26]. We like to put our results into this context. Herein we demonstrate that RE(amd)_3_ releases the RE component by selective thermolysis even in the absence of additional reducing agents. Microwave heating and employing suitable ILs and PC as inert reaction media proved to be crucial.

Ionic liquids are used as stabilizing reagents and as solvents in syntheses of nanoparticles [27–31]. In contrast to conventional stabilizers, such as surfactants or polymers, the ILs stabilize the particles via electrostatic and steric interactions without altering the surface properties [32–37]. In addition, they have a high thermal stability, negligible vapor pressure and are not flammable [38]. PC is an aprotic, highly dipolar solvent with low viscosity [39–40], low flammability and low toxicity [41].

## Results and Discussion

### Decomposition of RE(amd)_3_ and Eu(dpm)_3_

Thermogravimetric analysis (TGA) revealed decomposition of the rare-earth metal(III) tris(*N*,*N*′-diisopropyl-2-methylamidinate) (RE(amd)_3_; RE = Pr(III), Gd(III), Er(III) and tris(2,2,6,6-tetramethyl-3,5-heptanedionato)europium(III) (Eu(dpm)_3_) at temperatures between 160 and 230 °C (Table S1 and Figure S2, Supporting Information File 1). To keep the formation of by-products as low as possible and to achieve complete decomposition of the precursors, a temperature of 230 °C was selected on the basis of these TGA measurements for all microwave-assisted thermal NP syntheses. As reaction media we used the fluorous IL 1-butyl-3-methylimidazolium tetrafluoroborate ([BMIm][BF_4_]) and 1-butyl-3-methylimidazolium bis(trifluoromethylsulfonyl)imide ([BMIm][NTf_2_]) and the organic solvent propylene carbonate (PC) (Figure S3, Supporting Information File 1).

The rare-earth metal amidinates and Eu(dpm)_3_ were suspended under an argon atmosphere in dried IL or in PC. The compounds were decomposed by microwave irradiation for 20 min at a power of 50 W at a temperature of 230 °C (Scheme 1). The size distributions of the obtained nanoparticles were determined by transmission electron microscopy (TEM) and high-angle annular dark-field scanning transmission electron microscopy (HAADF-STEM). The crystalline phases and the absence of impurities (oxides) in RE-NPs and REF_3_-NPs were identified by powder X-ray diffraction (PXRD) and selected area electron diffraction (SAED). The results are summarized in Table 1.

**Table 1:** RE-/REF_3_-NPs sizes and size distributions.^a^

precursor	phase, identity of RE-/REF_3_-NPs^b^	TEM diameter [nm]^c^
[BMIm][BF_4_]		

Pr(amd)_3_	PrF_3_	11 ± 6
Eu(dpm)_3_	EuF_3_	23 ± 7
Gd(amd)_3_	GdF_3_	38 ± 17
Er(amd)_3_	ErF_3_	14 ± 5

[BMIm][NTf_2_]		

Pr(amd)_3_	—^d^	—^d^
Eu(dpm)_3_	—^d^	—^d^
Gd(amd)_3_	Gd^e^	1.5 ± 0.5
Er(amd)_3_	Er^e^	3.0 ± 0.5

PC		

Pr(amd)_3_	Pr^e^	2 ± 1
Eu(dpm)_3_	—^d^	—^d^
Gd(amd)_3_	Gd^e^	1.5 ± 0.5
Er(amd)_3_	Er^e^	5 ± 1

^a^1.0 wt % RE/REF_3_-NP/IL or in PC dispersions obtained by microwave irradiation with 50 W for 20 min at 230 °C. ^b^The phases of the nanoparticles were identified by PXRD and SAED. ^c^Average diameter and standard deviation σ. See Experimental section for TEM measurement conditions. ^d^No separated nanoparticles. ^e^No reflections in the PXRD.

### REF_3_-NPs from RE(amd)_3_ and Eu(dpm)_3_ in [BMIm][BF_4_]

The microwave-induced decomposition of the rare-earth metal amidinates RE(amd)_3_ with RE = Pr(III), Gd(III), Er(III) and Eu(dpm)_3_ in the fluorine-containing IL [BMIm][BF_4_] gave green (PrF_3_), white (EuF_3_, GdF_3_) and rose-coloured (ErF_3_) 1.0 wt % dispersions of REF_3_-NPs in IL.

Schmitz et al. synthesized REF_3_-NPs with RE = Pr, Eu, supported on different types of thermally reduced graphite oxide (TRGO) in [BMIm][BF_4_] [12]. The formation of REF_3_-NPs is due to the [BF_4_]^−^ anion in the IL [BMIm][BF_4_]. The [BF_4_]^–^ anion hydrolyzes or decomposes to fluoride F^−^ with small amounts of residual water in the IL (30 ppm) which is very difficult to remove from hydrophilic [BMIm][BF_4_] [12,42]. It is also known that the [BF_4_]^−^ anion decomposes to fluoride at high temperature [43]. According to ion chromatographic (IC) analysis, a fluoride source other than the [BF]^−^ anion can be excluded since the IL contains only a very small amount of fluoride ions (below 1 ppm, see Experimental section for IC analysis conditions) [44–45]. Alternatively, reactive metal atoms or metal clusters may also abstract fluoride from [BF_4_]^−^ anions. ILs are not only solvents but can also be reactants, e.g., [BMIm][PF_6_] in the synthesis of A_2_SiF_6_ nanoparticles (A = Li, Na, K, Rb, Cs) [46], in the synthesis of MF*_x_* nanoparticles (M = Mn, Fe, Co, Pr, Eu) from the decomposition of transition-metal amidinates in [BMIm][BF_4_] [12,42] or in the synthesis of metal-fluoride nanoparticles from metal acetate (hydrate) in ethylene glycol and an excess of [BMIm][BF_4_] [47].

**Figure 1:**
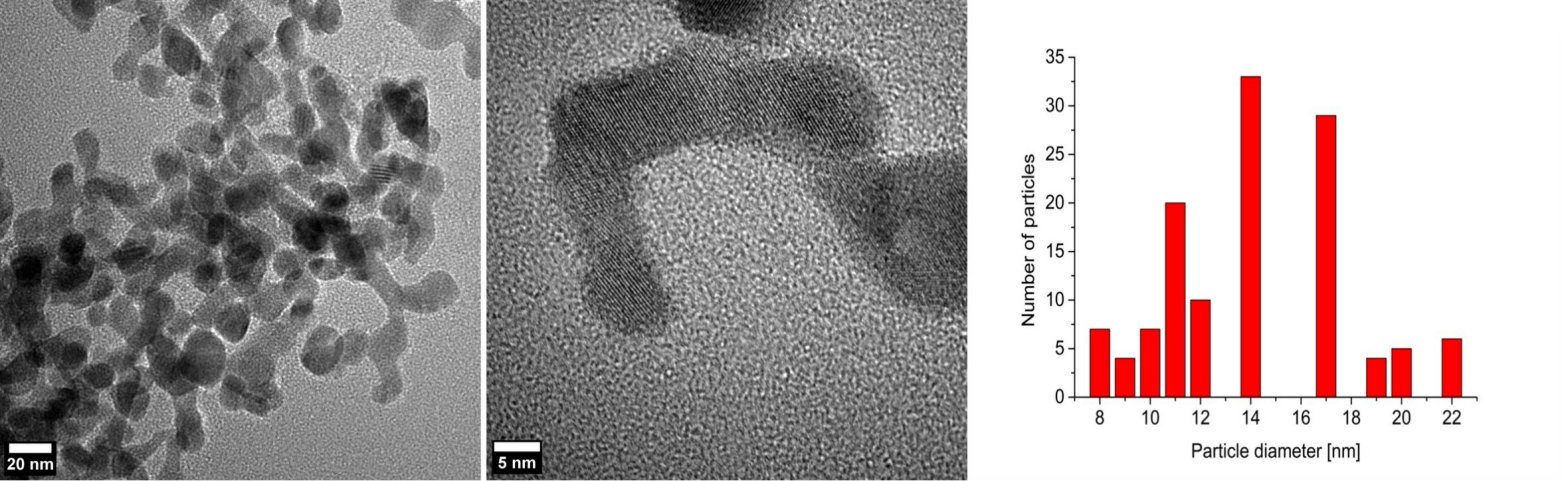
TEM images and particle size histogram (from 128 particles) of 1.0 wt % ErF_3_-NPs in [BMIm][BF_4_] from Er(amd)_3_. Here and in the other TEM histogram evaluations the size measurements were done assuming approximately spherical particles. Differentiation of the overlapping particles was done with magnified images and with the help of the fine contrast.

The sizes and size distributions of the REF_3_ nanoparticles were determined by HAADF-STEM (Figure S4a, Supporting Information File 1) and TEM (Figure 1 and Figures S5a and S6a, Supporting Information File 1) to values of 5–33 nm for PrF_3_, 6–35 nm for EuF_3_, 17–67 nm for GdF_3_ and 8–22 nm for ErF_3_. The average particle sizes are 11 ± 6 nm for PrF_3_, 23 ± 7 nm for EuF_3_, 38 ± 17 nm for GdF_3_, and 14 ± 5 nm for ErF_3_ (Table 1). STEM/TEM images show small, nearly spherical and partially agglomerated particles. Close-up TEM images show interference patterns for EuF_3_ and ErF_3_, which indicate crystallinity of the REF_3_-NPs (Figure 1 middle, and Figure S5a, Supporting Information File 1).

**Figure 2:**
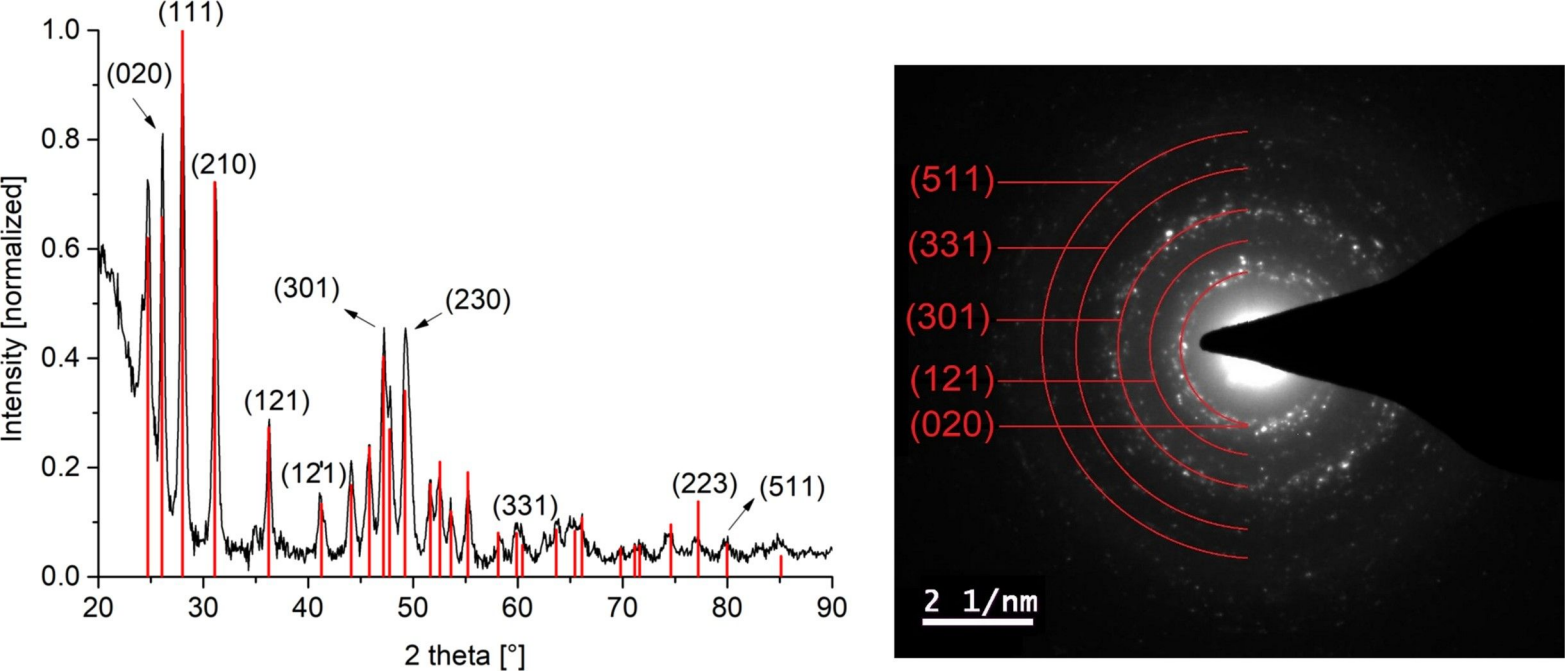
PXRD and SAED (ErF_3_ reference peaks in red from COD 4030804, orthorhombic structure with space group *Pnma*) of 1.0 wt % ErF_3_-NPs in [BMIm][BF_4_] from Er(amd)_3_.

The crystallinity of the particles was confirmed and the crystal phases were determined as pure rare-earth fluorides REF_3_ (RE = Pr(III), Eu(III), Gd(III), Er(III)) by powder X-ray diffractometry (PXRD) (Figure 2 and Figures S4b, S5b, S6b, Supporting Information File 1). In addition, the crystalline phases of GdF_3_ (Figure S6b, Supporting Information File 1) and ErF_3_-NPs (Figure 2) were assigned by selected area electron diffraction (SAED).

**Figure 3:**
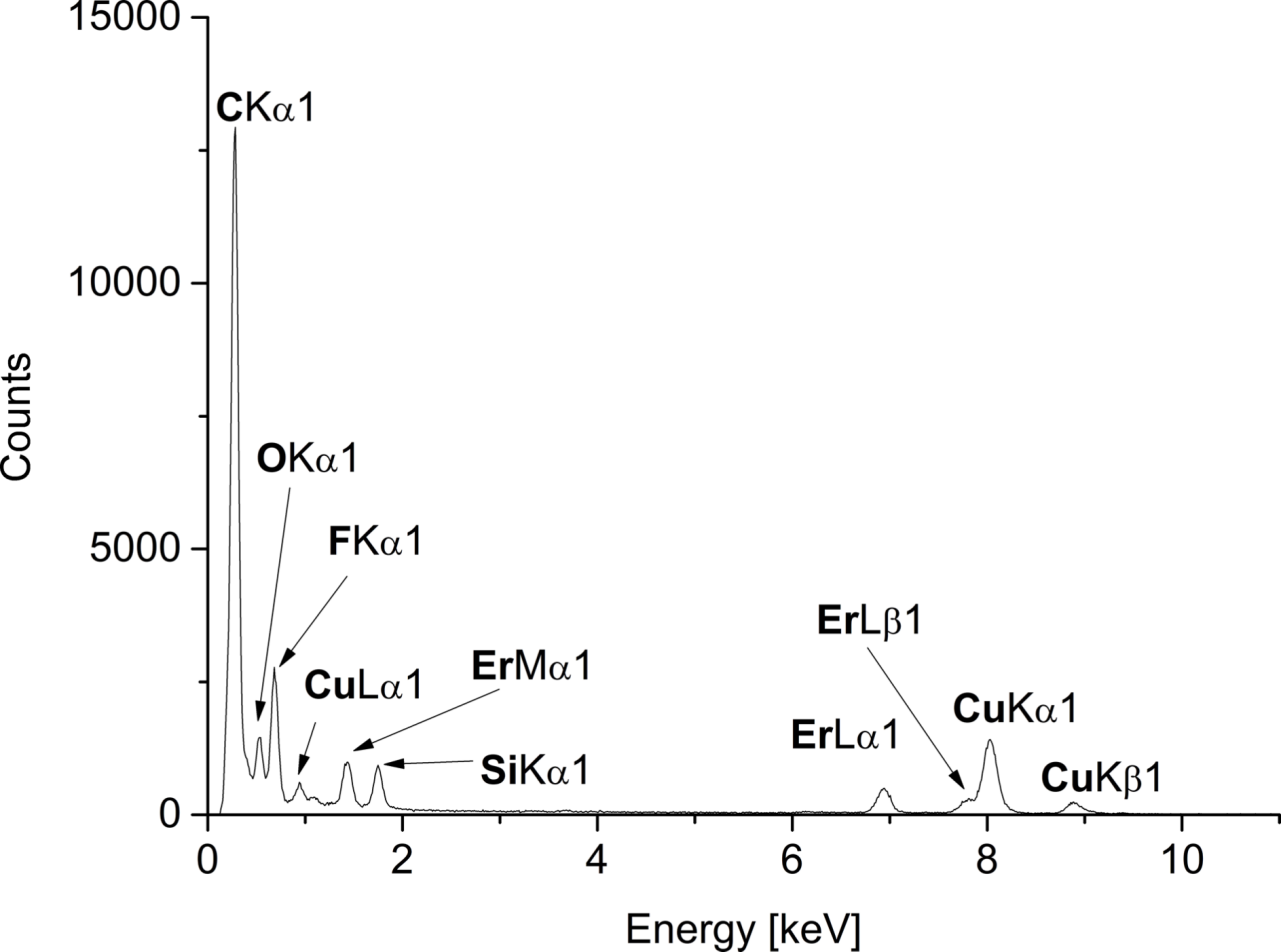
EDX of 1.0 wt % ErF_3_-NPs in [BMIm][BF_4_] from Er(amd)_3_.

The characterization was completed by energy-dispersive X-ray spectroscopy (EDX, in combination with TEM) for the qualitative element composition. EDX spectroscopy (Figure 3 and Figures S5b, S6c, Supporting Information File 1) show the expected signals for Eu, Gd or Er and fluoride besides the bands for carbon and copper of the carbon-coated copper grid. The oxygen peak can largely be attributed to air contamination when the sample was introduced into the TEM device. A quantification of fluoride against rare-earth metal was not done, because matching of the F Kα_1_ binding energy against the Lα_1_ or Lβ_1_ binding energies for Eu, Gd and Er is not very accurate.

**Figure 4:**
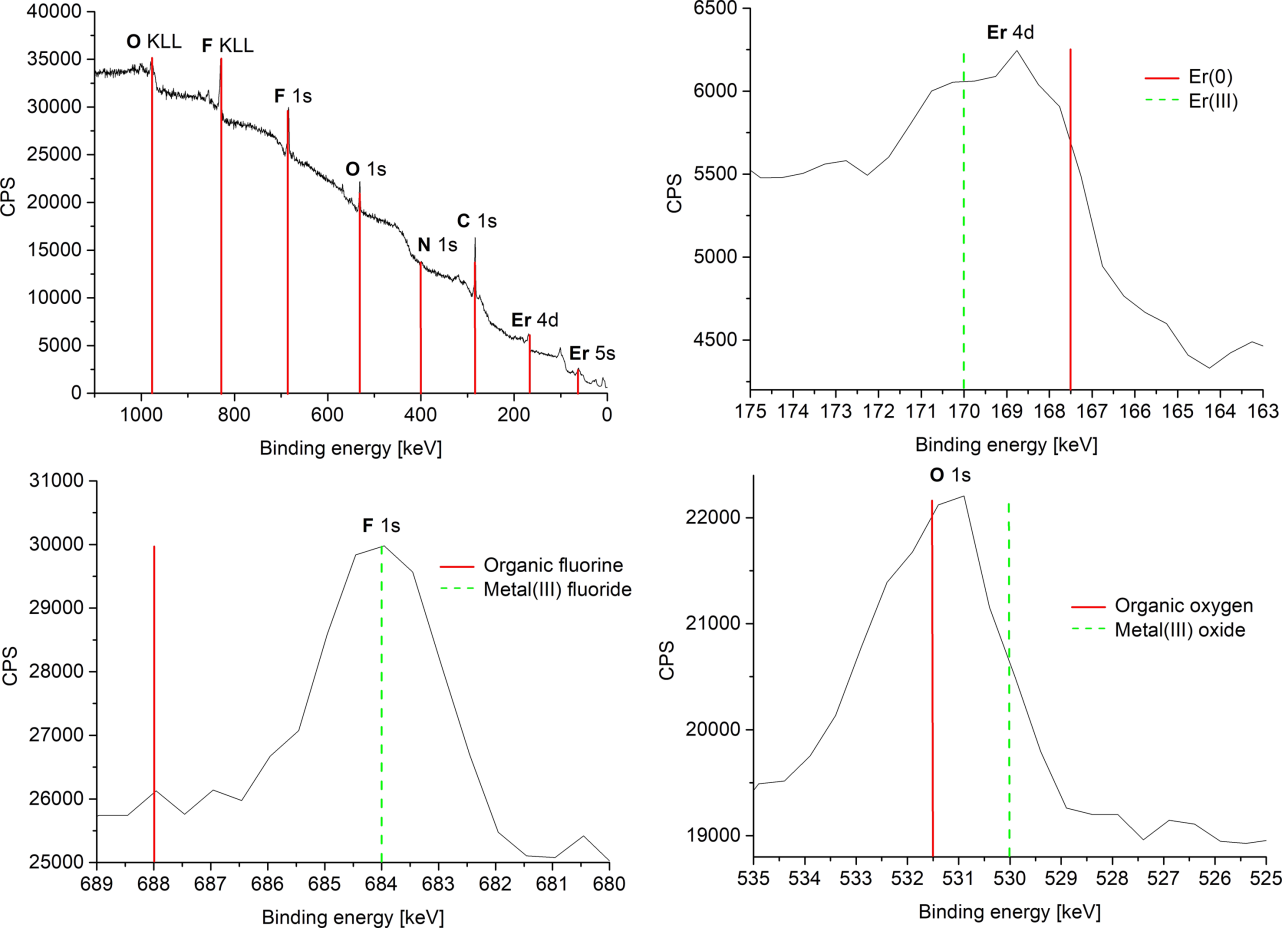
Overview and HR-XPS of 1.0 wt % ErF_3_-NPs in [BMIm][BF_4_] from Er(amd)_3_. The red and green bars are a guide to the eye on the binding-energy axis.

The measured oxidation state 3+ of the rare-earth metals in the fluorides was corroborated by high-resolution X-ray photoelectron spectroscopy (HR-XPS) (Figure 4 and Figures S4c, S5c, S6d, Supporting Information File 1) through comparison to the reported binding energies of metal(III) fluorides/oxides, metal(0) and organic fluorine/oxygen (Table 2) [48–49]. The measured metal and fluorine XPS values are in good agreement with the values of metal(III) fluorides that are given in literature and thereby exclude the formation of metal(0) and the presence of organic fluoride (from residual IL). In addition, the formation of metal oxides can be excluded, since the measured binding energies of oxygen match very well with the literature values of organic oxygen [48–49]. For EuF_3_, no oxygen peak was seen in the XPS analysis. Therefore, SAED and PXRD data in combination with HR-XPS exclude any contamination of the REF_3_-NPs with metal(III) oxides.

**Table 2:** Comparison of XPS binding energies in REF_3_-NP samples in [BMIm][BF_4_].

XPS signal	measured [eV]	metal(III) fluorides/oxides [eV] [48–49]	metal(0) and organic fluorine/oxygen [eV] [48–49]
PrF_3_			

Pr 3d_5/2_	934.5	933–936^a^	932
F 1s	686	684–685.5^a^	688–689
O 1s	533	529–530^a^	531.5–533

EuF_3_			

Eu 3d_5/2_	1135.5	1135^a^	1126
F 1s	685.5	684–685.5^a^	688–689
O 1s	no signal	529–530^a^	531.5–533

GdF_3_ [50]			

Gd 3d_3/2_	1220	1220^a^	1218
Gd 3d_5/2_	1188	1188^a^	1186
Gd 4d	142.5	144^a^	140
F 1s	684	684–685.5^a^	688–689
O 1s	531.5	529–530^a^	531.5–533

ErF_3_			

Er 4d	169	170^a^	167.5
F 1s	684	684–685.5^a^	688–689
O 1s	531	529–530^a^	531.5–533

^a^Entry corresponds to the measured experimental value.

**Figure 5:**
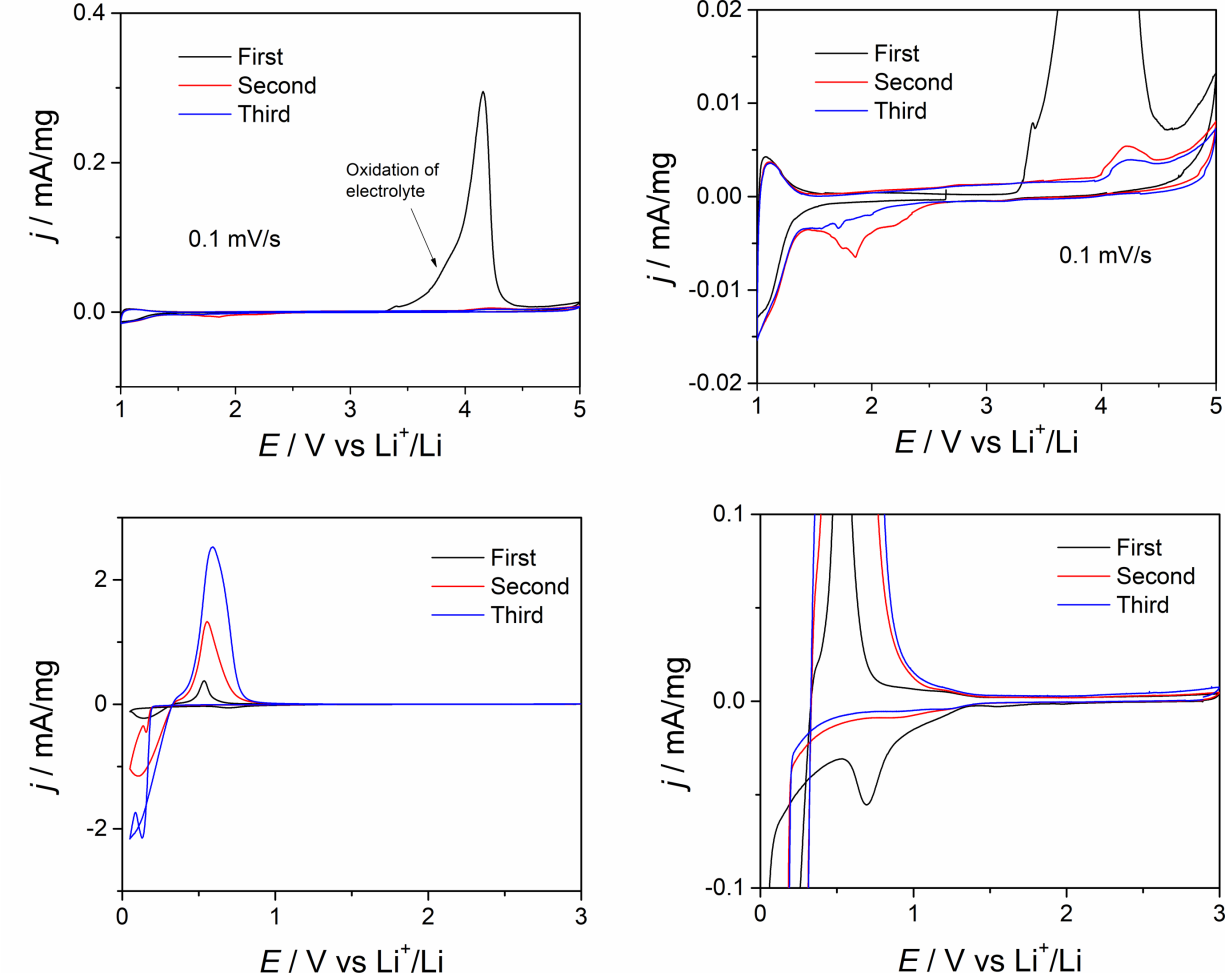
Cyclic voltammetry of a half-cell with ErF_3_ as working electrode and lithium foil as counter electrode at the different cut-off potentials. The figures at the right represent magnifications of the two left figures.

Metal fluorides are used, for example, as cathode materials in lithium-ion batteries [6]. The lithium-ion battery is one of the most important rechargeable energy storage devices in modern electrical appliances such as mobile phones and laptops, but also in electric and hybrid vehicles [51]. The increasing performance of modern lithium-ion batteries is of great interest in current research [52–54]. Grey et al. showed that the use of FeF_2_ nanoparticles as electrode material leads to a significant increase in the performance of the batteries compared to the macroscopic LiFeF_3_ [55]. Therefore, we investigated the electrochemical properties of ErF_3_-NPs by galvanostatic charge/discharge profiles (Figure 5).

Until now there has been no report on ErF_3_ applied as electrode material for Li^+^/Li. Cyclic voltammetry was performed to address general aspects of the redox behaviour (Figure 5). In the range from 1.0 to 5.0 V, during the reduction, a reduction process takes place starting at 1.5 V, which may be attributed to the transformation of Er^3+^ to Er^2+^, which can be reversibly oxidized. During the oxidation process, oxidation of the electrolyte was observed, starting around 3.4 V. Hence, such standard electrolytes cannot be applied to this redox couple. In the range from 1.0 to 0.05 V, there is an overpotential electrodeposition process of Al^3+^, stemming from the Al collector, indicating that the used potential cannot be lower than 0.2 V. The open-circuit potential (OCP) of the cell is around 2.7 V vs Li^+^/Li.

The standard electrode potential values of the involved redox couples are: Li^+^ + e^−^ → Li (−3.04 V vs SHE), Er^3+^ + 3e^−^ → Er (−2.33 V vs SHE; 0.71 V vs Li^+^/Li) and Er^2+^ + 2e → Er (−2.0 V vs SHE; 1.04 V vs Li^+^/Li). In conclusion, ErF_3_ does not exhibit reversible redox behaviour using common electrolytes, and thus more elaborate experimental effort is needed, including changing the potential range or electrolyte, or choosing Cu instead of Al for the current collector.

### RE-NPs from RE(amd)_3_ in [BMIm][NTf_2_]

Compared to the IL [BMIm][BF_4_], which can act as a fluoride source, the hydrophobic IL [BMIm][NTf_2_] with C–F bound fluorine can be expected to be a more inert reaction medium. Further, this hydrophobic IL could be more readily dried to residual water levels (determined by Karl Fischer titration) of below 10 ppm (cf. 30 ppm for [BMIm][BF_4_]). The rare-earth metal amidinates RE(amd)_3_ with RE = Pr(III), Gd(III), Er(III) and Eu(dpm)_3_ were suspended in [BMIm][NTf_2_] and decomposed by microwave irradiation for 20 min at a temperature of 230 °C (Scheme 1 and Figure S3, Supporting Information File 1).

**Figure 6:**
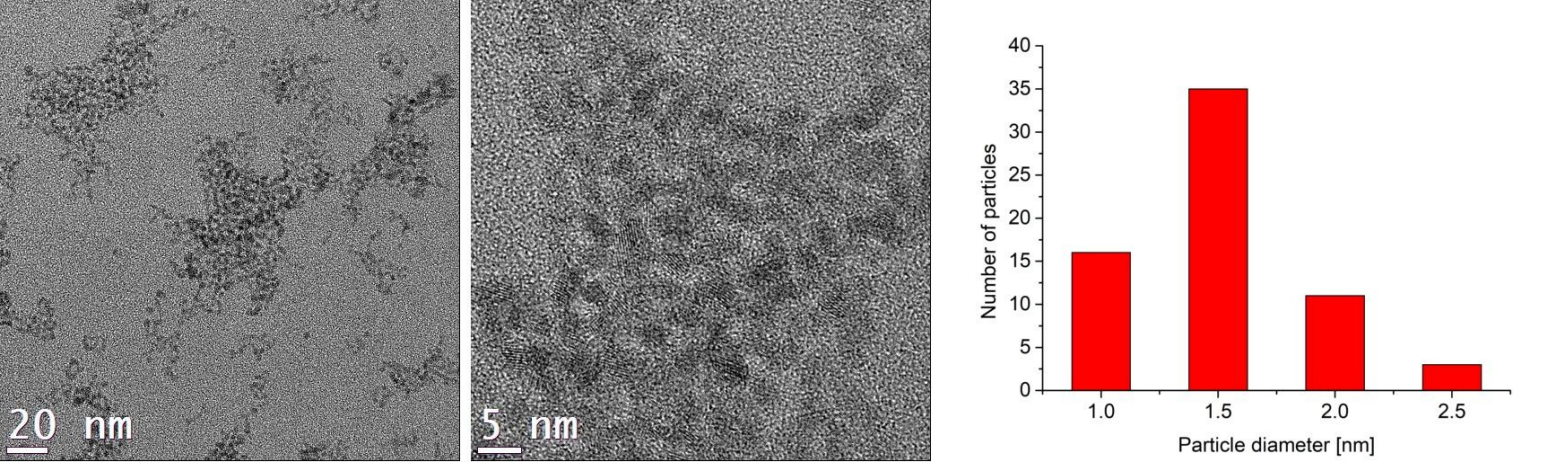
TEM images and particle size histogram of 1.0 wt % Gd-NPs in [BMIm][NTf_2_] from Gd(amd)_3_.

Rare-earth metal nanoparticles (RE-NPs) were obtained for RE = Gd and Er. For Pr(amd)_3_ and Eu(dpm)_3_ no particles were seen in TEM investigations. The size and size distribution of the Gd-NPs and Er-NPs were determined by TEM (Figure 6 and Figure S7a, Supporting Information File 1) to values of 1.0–2.5 nm for Gd (average diameter of 1.5 ± 0.5 nm) and 2.0–3.5 nm for Er (average diameter 3.0 ± 0.5 nm) (Table 1). A close-up of the TEM images shows interference patterns, indicating crystallinity of the RE-NPs.

**Figure 7:**
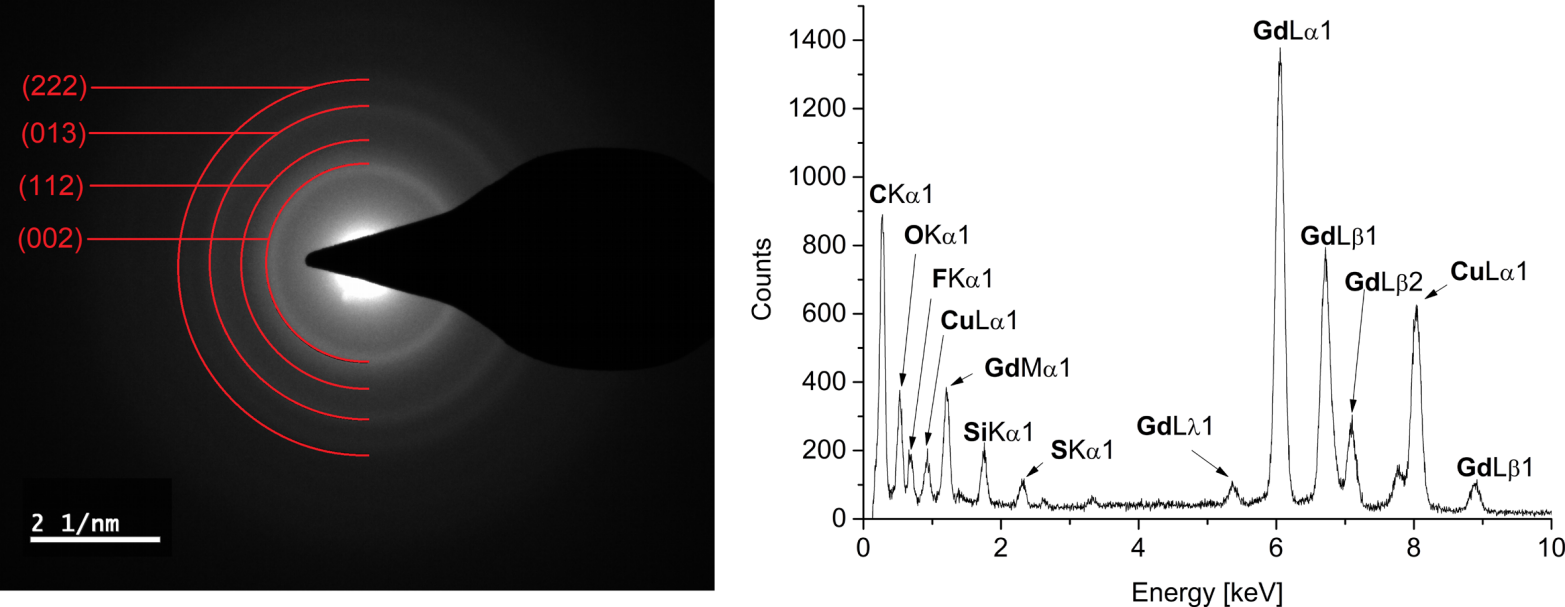
SAED (Gd reference peaks in red from COD 1522526, cubic crystal system with space group *Im*−3*m*) and EDX of 1.0 wt % Gd-NPs in [BMIm][NTf_2_] from Gd(amd)_3_.

The crystallinity of the RE-NPs was confirmed by SAED and gave the expected reflections for elemental Gd and Er (Figure 7 and Figure S7b, Supporting Information File 1). Due to the very small size of the Gd and Er particles a meaningful PXRD pattern could not be obtained.

Characterization by EDX (Figure 7 and Figure S7b, Supporting Information File 1) gave the expected bands for Gd and Er. The small fluorine and sulfur peaks are due to residual IL around the nanoparticles (Figure S3, Supporting Information File 1). We suggest that the residual IL coverage of the RE-NPs also prevents their oxidation during the short air contact upon transfer from a Schlenk flask into the TEM and XPS.

**Figure 8:**
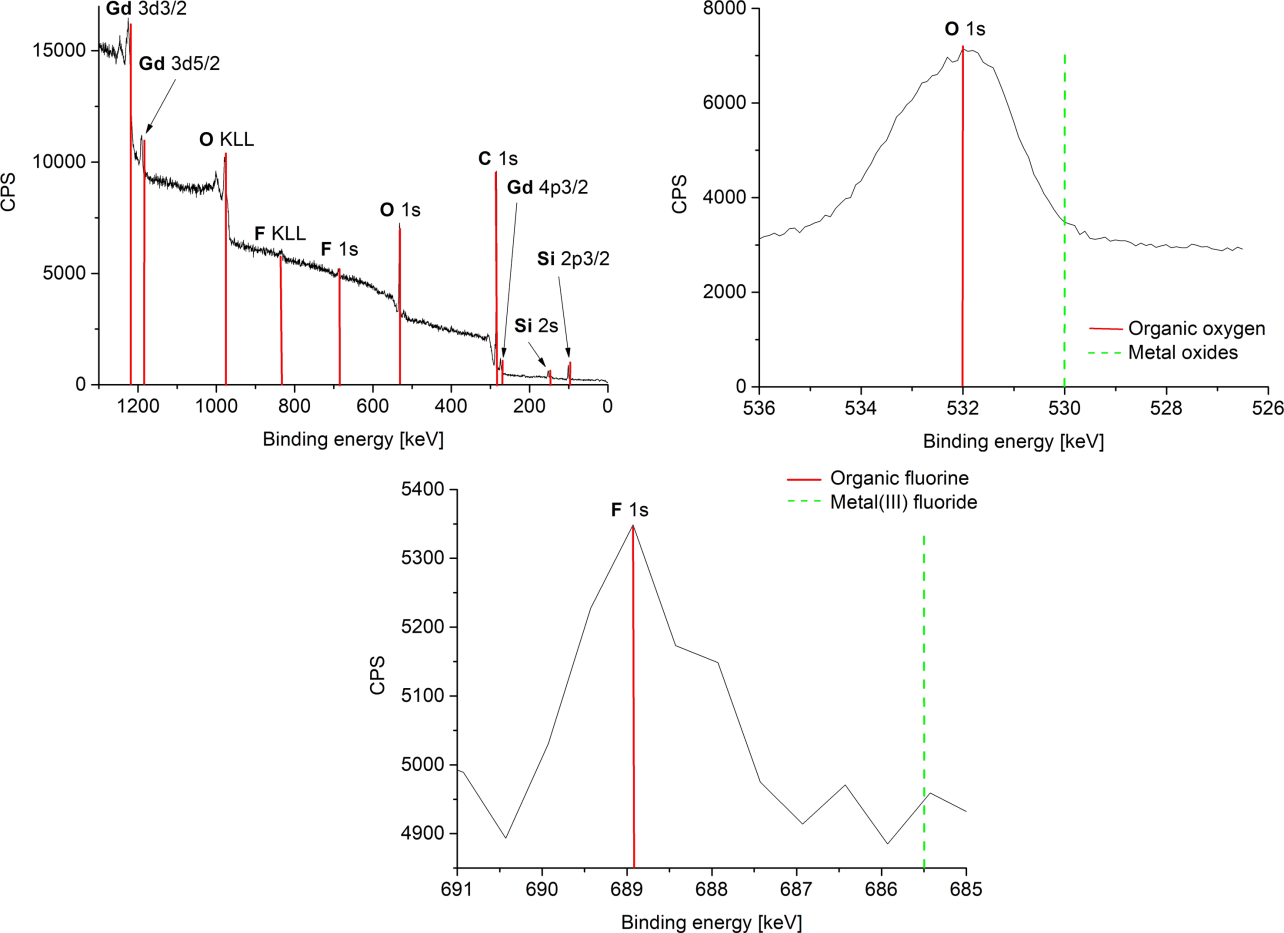
Overview and HR-XPS of 1.0 wt % Gd-NPs in [BMIm][NTf_2_] from Gd(amd)_3_. The red and green bars are a guide to the eye on the binding-energy axis.

The oxidation state zero of gadolinium and erbium, i.e., the formation of Gd(0) and Er(0) metal NPs were indirectly supported by the measured XPS binding energies of oxygen and fluorine (Figure 8, Table 3 and Figure S7c, Supporting Information File 1). An assignment of the measured RE (RE = Gd, Er) binding energies to metal(0) or metal(III) oxide was not possible because the binding energies are shifted strongly due to the very small size of the NPs. The measured XPS binding energies of oxygen and fluorine match very well with the literature values of organic oxygen and organic fluorine and thereby exclude the formation of RE(III) oxide or RE(III) fluoride for RE = Gd(0) and Er(0) [48–49]. Thus, HR-XPS data in combination with SAED exclude any significant contamination of Gd(0)-NPs and Er(0)-NPs with oxides or fluorides. We note that the amount of metal oxides is below the detection limit and that small amounts of water in the ILs of up to 30 ± 18 ppm for [BMIm][BF_4_] does not lead to a detectable formation of metal oxides. The water amount in the less hydrophilic IL [BMIm][NTf_2_] was even lower (10 ppm).

**Table 3:** Comparison of XPS binding energies in RE-NP samples in [BMIm][NTf_2_].

XPS signal	measured [eV]	metal(III) oxides or metal(III) fluorides [eV] [48–49]	metal(0) and organic oxygen or organic fluorine [eV] [48–49]
Gd(0)-NPs [50]			

Gd 3d_3/2_	1225	1220	1218
Gd 3d_5/2_	1191.5	1188	1186
O 1s	532	529–530	531.5–533^a^
F 1s	689	684–685.5	688–689^a^

Er(0)-NPs			

Er 4d	174	170	167.5
O 1s	534.5	529–530	531.5–533^a^
F 1s	690	684–685.5	688–689^a^

^a^Entry corresponds to the measured experimental value.

### RE-NPs from RE(amd)_3_ in PC

In addition to the ILs [BMIm][BF_4_] and [BMIm][NTf_2_], the organic and dried solvent PC (<10 ppm water content) (Figure S3, Supporting Information File 1) was used for NP syntheses. The rare-earth metal amidinates RE(amd)_3_ with RE = Pr(III), Gd(III), Er(III) and Eu(dpm)_3_ were suspended in PC and decomposed in the microwave reactor for up to 20 min at 230 °C (Scheme 1).

**Figure 9:**
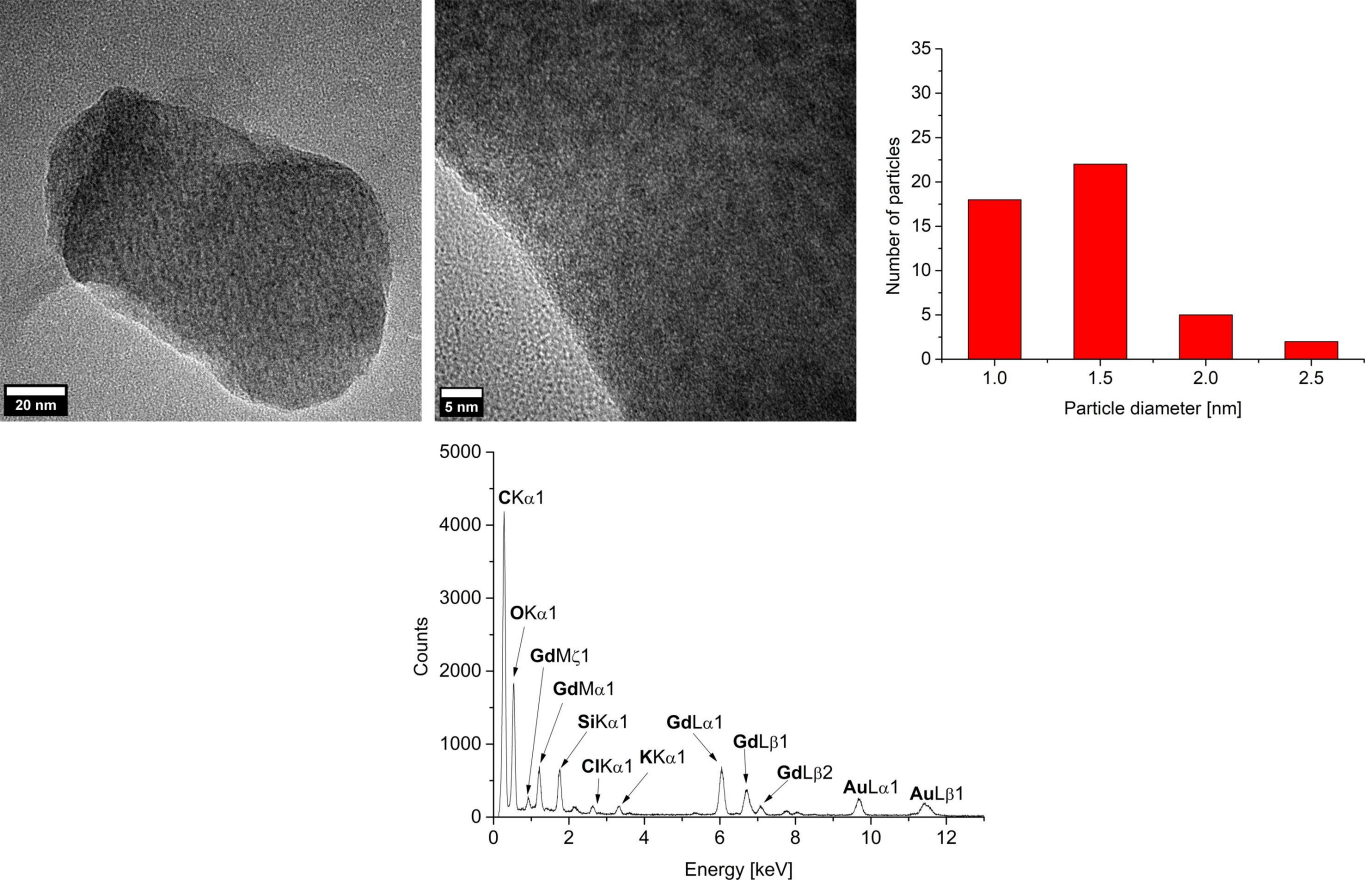
TEM images, particle-size histogram and EDX of 1.0 wt % Gd-NPs in PC from Gd(amd)_3_.

The microwave-assisted thermal decomposition gave rare-earth metal nanoparticles (RE-NPs). Again, for Eu(dpm)_3_ no particles were seen in TEM investigations. The size and size distribution of the RE-NPs were determined by TEM (Figure 9 and Figures S8, S9a, Supporting Information File 1) to values of 1.5–3.5 nm for Pr, 1.0–2.5 nm for Gd and 4.0–7.0 nm for Er giving average diameters of 2 ± 1 nm for Pr, 1.5 ± 0.5 nm for Gd and 5 ± 1 nm for Er (Table 1). Interference patterns for Pr-NPs and Gd-NPs in close-up TEM images (Figure 9 top and Figure S8, Supporting Information File 1) indicate crystallinity of the RE-NPs. For the crystalline Er-NPs SAED gave the expected reflections of elemental erbium (Figure S9b, Supporting Information File 1). The very small size of the particles failed to yield meaningful PXRD patterns.

EDX (Figure 9 and Figure S9b, Supporting Information File 1) gave the expected bands for Gd and Er. No fluorine was detected by EDX analysis. The oxygen peak can be attributed mainly to air contamination when the sample was introduced into the TEM device.

**Figure 10:**
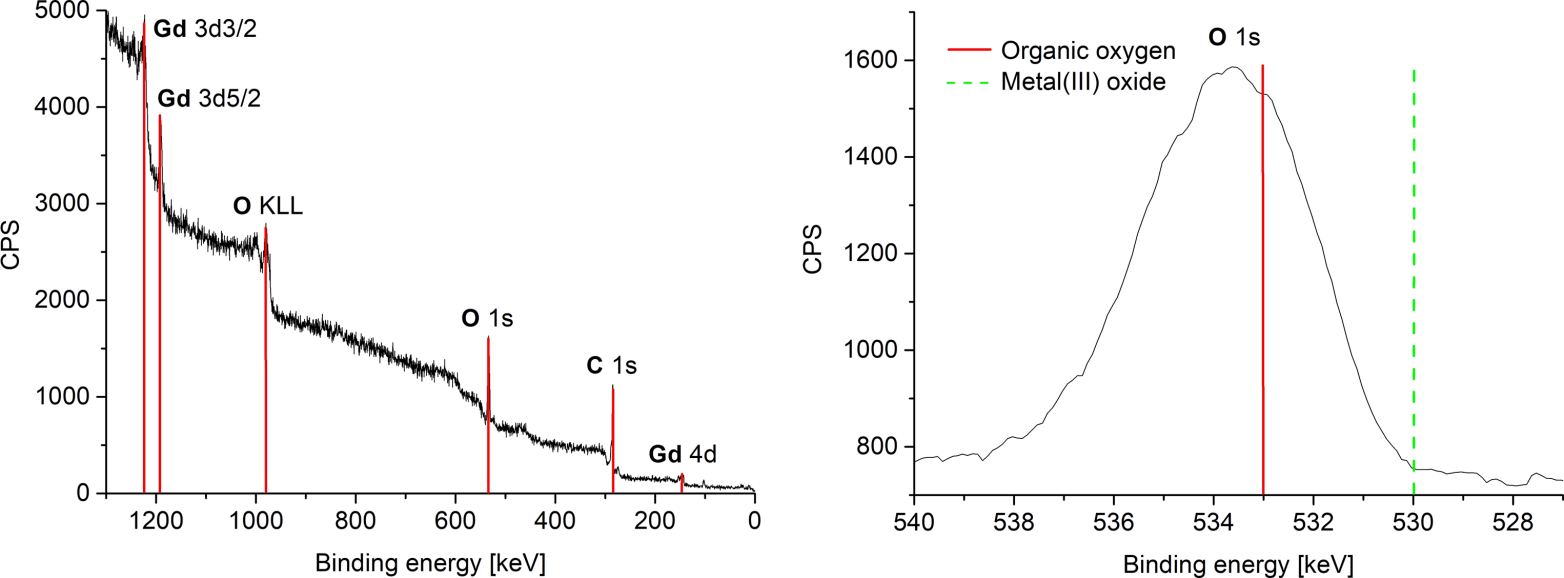
HR-XPS of 1.0 wt % Gd-NPs in PC from Gd(amd)_3_. The red and green bars are a guide to the eye on the binding-energy axis.

Again, the formation of Pr(0), Gd(0) and Er(0) metal NPs were indirectly supported by the measured XPS binding energies of oxygen (Figure 10, Table 4 and Figures S8b and S9c, Supporting Information File 1). The measured RE (RE = Pr, Gd, Er) binding energies had again shifted due the small NP size so that no clear assignment to metal(0) or metal(III) could be made. The measured XPS binding energies of oxygen are in very good agreement with the binding energies of organic oxygen that are given in literature and thereby exclude the formation of RE(III) oxide for RE = Pr(0), Gd(0) and Er(0) [48–49]. Understandably, the non-fluorous solvent PC (Figure S3, Supporting Information File 1) gave no signals for fluorine.

**Table 4:** Comparison of XPS binding energies in RE-NPs samples in PC.

XPS signal	measured [eV]	metal(III) oxides or metal(III) fluorides [eV] [48–49]	metal(0) and organic oxygen or organic fluorine [eV] [48–49]
Pr(0)-NPs			

Pr 3d_5/2_	934	933–936	932
O 1s	532	529–530	531.5–533^a^
F 1s	no signal	684–685.5	688–689^a^

Gd(0)-NPs [50]			

Gd 3d_3/2_	1223	1220	1218
Gd 3d_5/2_	1191	1188	1186
Gd 4d	146	144	140
O 1s	533.5	529–530	531.5–533^a^
F 1s	no signal	684–685.5	688–689^a^

Er(0)-NPs			

Er 4d	172	170	167.5
O 1s	532.5	529–530	531.5–533^a^
F 1s	no signal	684–685.5	688–689^a^

^a^Entry corresponds to the measured experimental value.

## Conclusion

Microwave-assisted thermal decomposition [56–58] using the rare-earth metal(III) coordination compounds tris(*N*,*N*′-diisopropyl-2-methyl-amidinate) RE(amd)_3_ (RE = Pr(III), Gd(III), Er(III)) and tris(2,2,6,6-tetramethyl-3,5-heptanedionato)europium(III) (Eu(dpm)_3_) as precursor compounds yield REF_3_-NPs in the IL [BMIm][BF_4_] for RE = Pr, Eu, Gd and Er and very small RE-NPs in the IL [BMIm][NTf_2_] and in propylene carbonate (PC) for RE = Pr, Gd and Er. The phase purity and the absence of oxide impurities was proven by powder X-ray diffraction (PXRD), selected area electron diffraction (SAED) and high-resolution X-ray photoelectron spectroscopy (HR-XPS). To the best of our knowledge, there have been so far no reports on the synthesis of non-oxidized nanoparticles of any rare-earth element by soft-wet chemical routes from metal-organic precursors. However, access to a simple, reproducible and scalable method to obtain RE-NPs in solution will be the key for developing the nano-chemistry of non-oxide (and non-fluoride) RE materials. Our results on praseodymium, gadolinium and erbium nanoparticles derived from microwave-assisted thermolysis of the respective metal amidinates RE(amd)_3_ as precursors may open up new avenues for applications of pure rare-earth metal nanoparticles and nanomaterials derived from these. In particular we are aiming to study the nano-alloying of RE-NPs with late transition metals M and study the catalytic properties of the obtained intermetallic M/RE-NPs in extension of our previous work on Ni/Ga nanophases derived from organometallic precursors by co-thermolysis in ILs and PC [59].

## Experimental

All synthesis experiments were carried out with Schlenk techniques under nitrogen or argon since the amidinates are hygroscopic and air sensitive. Propylene carbonate (PC) was obtained from Sigma Aldrich (99.7%) and was dried under high vacuum (1.0 Pa) for a few days. The water content of PC measured by using coulometric Karl Fischer titration (ECH/Analytik Jena AQUA 40.00) was less than 10 ppm. Methyllithium, 1,3-diisopropylcarbodiimide (>99%) and praseodymium(III) chloride (>99%) were purchased from Sigma-Aldrich and used without further purification. Tris(2,2,6,6-tetramethyl-3,5-heptanedionato)europium(III) (>99%) was purchased from Alfa Aesar and was dried under high vacuum (10^−3^ mbar). 1-chlorobutane (>99%) and 1-methylimidazole (>99%) were obtained from Sigma-Aldrich and purified by fractional distillation, then dried over 4 Å molecular sieves for several days.

The syntheses of the rare-earth metal amidinates (RE(amd)_3_, RE = Gd, Er, Pr) were performed according to literature procedures [60–63]. The rare-earth metal amidinates RE(amd)_3_ were synthesized by an insertion reaction of methyl lithium into 1,3-diisopropylcarbodiimide in THF. The resulting lithium amidinate solution was reacted with the RE halides in a salt metathesis reaction.

The ionic liquids (ILs) [BMIm][BF_4_] and [BMIm][NTf_2_] were synthesized by reacting 1-methylimidazole with 1-chlorobutane to yield [BMIm][Cl]. The [BMIm][Cl] reacted with HBF_4_ or LiNTf_2_ to give [BMIm][BF_4_] or [BMIm][NTf_2_] [64]. The IL was dried under ultra-high vacuum (10^−7^ mbar) at 70 °C for several days. The characterization was carried out by ^1^H and ^13^C NMR. Quantitative anion exchange and, thus, IL purity was assessed by ion chromatography (Dionex ICS-1100, with IonPac^®^ AS22, 4 × 250 mm column) to be >99% for both ILs. The water content by coulometric Karl Fischer titration was less than 10 ppm for [BMIm][NTf_2_] and 30 ± 18 ppm for [BMIm][BF_4_].

**Synthesis procedures of rare-earth metal nanoparticles (RE-NPs) and rare-earth fluoride nanoparticles (REF_3_-NPs)** were based on previous literature [12]. All syntheses were carried out under inert conditions. The rare-earth metal amidinate precursors/[Eu(dpm)_3_] were suspended at room temperature in the dried ILs [BMIm][BF_4_], [BMIm][NTf_2_] or in PC. In contrast to the PrF_3_- and EuF_3_-NPs syntheses of Schmitz et al. [12], the rare-earth metal amidinate precursors and [Eu(dpm)_3_] were decomposed by microwave irradiation (CEM, Discover) for 20 min at a power of 50 W to a temperature of 230 °C (cf. 15 min, 50 W, 220 °C). The mass of the metal was adjusted to obtain 1.0 wt % NPs in IL or PC dispersion.

**HR-X-ray photoelectron spectroscopy:** HR-XPS (ESCA) measurements were performed with a Fisons/VG Scientific ESCALAB 200X xp-spectrometer, operating at 70–80 °C, a pressure of 7.0 × 10^−9^ mbar and a sample angle of 33°. Spectra were recorded using polychromatic Al Kα excitation (11 kV, 20 mA) and an emission angle of 0°. Calibration of the XPS was carried out by recording spectra with Al Kα X-rays from clean samples of copper, silver and gold at 50 eV and 10 eV pass energy and comparison with reference values.

**Powder X-ray diffraction:** PXRD data were measured at ambient temperature on a Bruker D2-Phaser using a flat sample holder and Cu Kα radiation (λ = 1.54182 Å, 35 kV). Samples had been precipitated with ethanenitrile from the NP/IL dispersion and washed several times with ethanenitrile. PXRDs were measured for 1 h. Small shifts in PXRD patterns are not uncommon for nanoparticles. A number of effects can be considered for such shifts including the range of stoichiometric composition, partly inhomogeneous element distribution, defects such as stacking and twin faults and nanosized crystalline domains being much smaller than the bulk reference material causing lattice contraction or expansion and strain [65–69].

**Transmission electron microscopy:** TEM was performed with a FEI Tecnai G2 F20 electron microscope operated at 200 kV accelerating voltage [70]. Conventional TEM images were recorded with a Gatan UltraScan 1000P detector. High-angle annular dark-field scanning transmission electron microscopy (HAADF-STEM) as shown in Figure S4a (Supporting Information File 1) was also performed with this microscope. TEM samples were prepared by drop-casting the diluted material on 200 μm carbon-coated copper grids or gold grids. The size distribution was determined manually or with the aid of the Gatan Digital Micrograph software from at least 50 (if not stated otherwise) individual particles. EDX spectra for composition analysis were recorded with the same instrument using an exposure time of 3 min.

**Selected area electron diffraction:** SAED patterns (Figures S4 and S6, Supporting Information File 1) have been recorded with an FEI Titan 80-300 TEM [71], operated at 300 kV accelerating voltage. The area selection was achieved with a round aperture placed in the first intermediate image plane with a corresponding diameter of 0.64 µm in the object plane. For each acquisition a sample region with a significant amount of material was placed inside the aperture. The objected was illuminated with wide-spread parallel beam obtaining focused diffraction patterns. The diffraction images were calibrated with Debye–Scherrer patterns recorded from a gold reference sample (S106, Plano GmbH, Wetzlar, Germany).

**Thermogravimetric analysis:** TGA was performed with Netzsch TG 209 F3 Tarsus equipped with an Al crucible by using a heating rate of 10 K·min^−1^.

**Electrochemical measurements:** The ErF_3_ working electrodes were prepared by coating an (*N*-methyl pyrrolidone)-based slurry composed of 75 wt % ErF_3_, 15 wt % conductive agents (Super P active carbon from Temical) and 10 wt % binder (PVDF) on a current collector (aluminium foil). A half-cell was assembled in an Ar-filled glovebox, with lithium foil as a counter electrode and 1 M LiPF_6_ in ethylene carbonate–ethyl methyl carbonate (50:50) as the electrolyte. The cyclic voltammetry (CV) data of these half-cells were collected utilizing an electrochemical workstation (Autolab 302) with different cut-off potentials.

## Supporting Information

Supporting Information contains: thermogravimetric analysis, TGA of rare-earth metal amidinates and Eu(dpm)_3_, structural formulas of the ionic liquids (ILs) and propylene carbonate (PC), TEM images, particle size histogram, PXRD, SAED, EDX and XPS of REF_3_-NPs, TEM images, particle size histogram, SAED, EDX and XPS of Er-NPs in [BMIm][NTf_2_], TEM images, particle size histogram, SAED, EDX and XPS of RE-NPs in PC.

File 1 Additional experimental data.
