# Supplementary material for: Synthesis of rare-earth metal and rare-earth metal-fluoride nanoparticles in ionic liquids and propylene carbonate
Source: Beilstein J Nanotechnol. 2018 Jun 28;9:1881–94. doi: 10.3762/bjnano.9.180 (PMC6036975; doi:10.3762/bjnano.9.180)
Supplement: File 1 — Additional experimental data. [file Beilstein_J_Nanotechnol-09-1881-s001.pdf]

Supporting Information

for

Synthesis of rare-earth metal and rare-earth metal-fluoride nanoparticles in ionic liquids and propylene carbonate

Marvin Siebels[1], Lukas Mai[2], Laura Schmolke[1], Kai Schütte[1], Juri Barthel[3], Junpei Yue[4], Jörg Thomas[5], Bernd M. Smarsly[4], Anjana Devi[2], Roland A. Fischer[6] and Christoph Janiak*[1]

Address: [1]Institut für Anorganische Chemie und Strukturchemie, Heinrich-Heine-Universität Düsseldorf, 40204 Düsseldorf, Germany. Fax: +49-211-81-12287; Tel: +49-211-81-12286; [2]Inorganic Materials Chemistry, Ruhr-Universität Bochum, 44801 Bochum, Germany; [3]Gemeinschaftslabor für Elektronenmikroskopie RWTH-Aachen, Ernst Ruska-Centrum für Mikroskopie und Spektroskopie mit Elektronen, 52425 Jülich, Germany; [4]Physikalisch-Chemisches Institut, Justus-Liebig-Universität Gießen, 35392 Gießen, Germany; [5]Department Structure and Nano-/Micromechanics of Materials, Max-Planck-Institut für Eisenforschung GmbH, 40237 Düsseldorf, Germany and [6]Department of Chemistry, Technische Universität München, 85748 Garching, Germany

Email: Christoph Janiak* - janiak@uni-duesseldorf.de

* Corresponding author

Additional experimental data

Content

Thermogravimetric analysis, TGA of rare earth amidinates and $Eu(dpm)_3$

Structural formulas of the ionic liquids (ILs) and propylene carbonate (PC)

TEM images, particle size histogram, PXRD, SAED, EDX and XPS of REF_3-NPs

TEM images, particle size histogram, SAED, EDX and XPS of Er-NPs in [BMIm][NTf_2]

TEM images, particle size histogram, SAED, EDX and XPS of RE-NPs in PC

Thermogravimetric analysis, TGA of rare earth amidinates and $Eu(dpm)_3$

Table S1: Thermogravimetric analysis of the rare earth metal amidinates and $Eu(dpm)_3$[a].

Precursor	Decomposition temperature [°C]	Δm_{TGA} (exptl/calcd)
**$Pr(amd)_3$**	160	66/70
**$Eu(dpm)_3$**	220	89/79
**$Gd(amd)_3$**	230	90/74
**$Er(amd)_3$**	220	82/72

[a] See thermogravimetric diagrams in Figure S2.

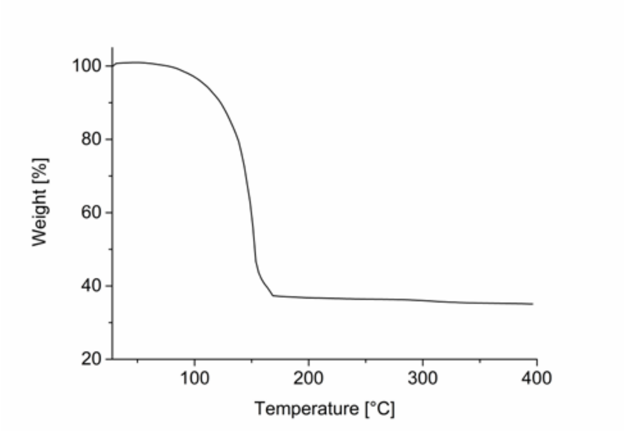

Figure S2a: TGA $Pr(amd)_3$, 25–400 °C, 5 K/min.

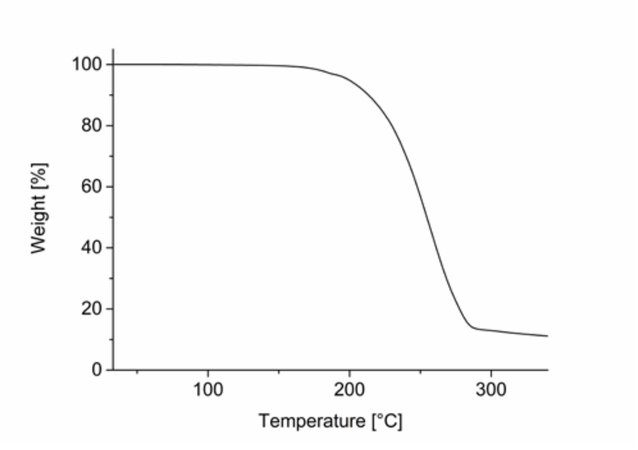

Figure S2b: TGA $Eu(dpm)_3$, 25–350 °C, 5 K/min.

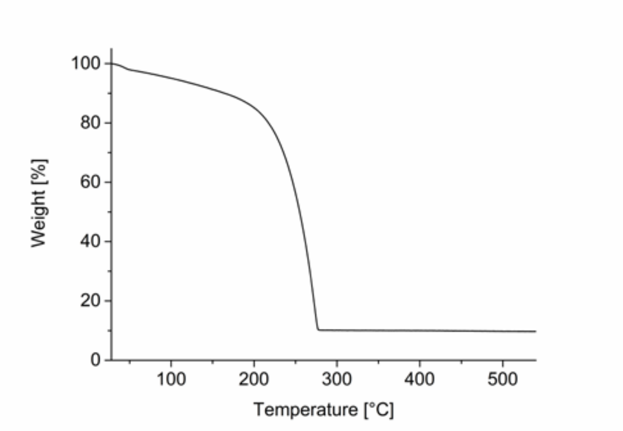

Figure S2c: TGA Gd(amd)$_3$, 25–550 °C, 5 K/min.

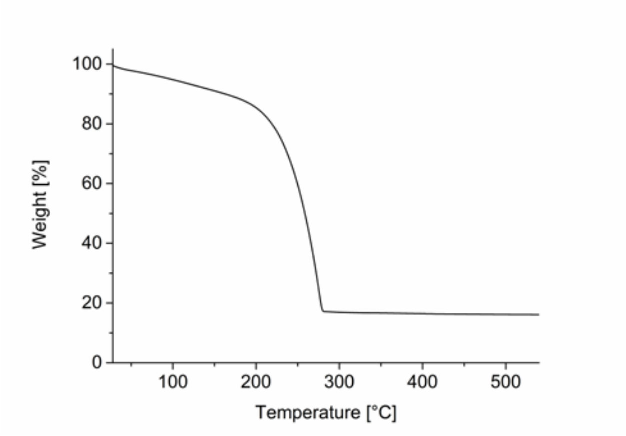

Figure S2d: TGA Er(amd)$_3$, 25–550 °C, 5 K/min.

Structural formulas of the ionic liquids (ILs) and propylene carbonate (PC)

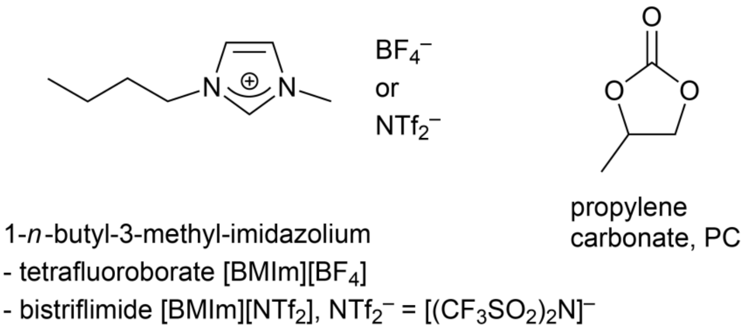

Figure S3: Ionic liquids (ILs) and propylene carbonate (PC) used as reaction media and stabilizer for NP syntheses.

TEM images, particle size histogram, PXRD, SAED, EDX and XPS of REF_3-NPs

PrF_3-NPs in [BMIm][BF_4]

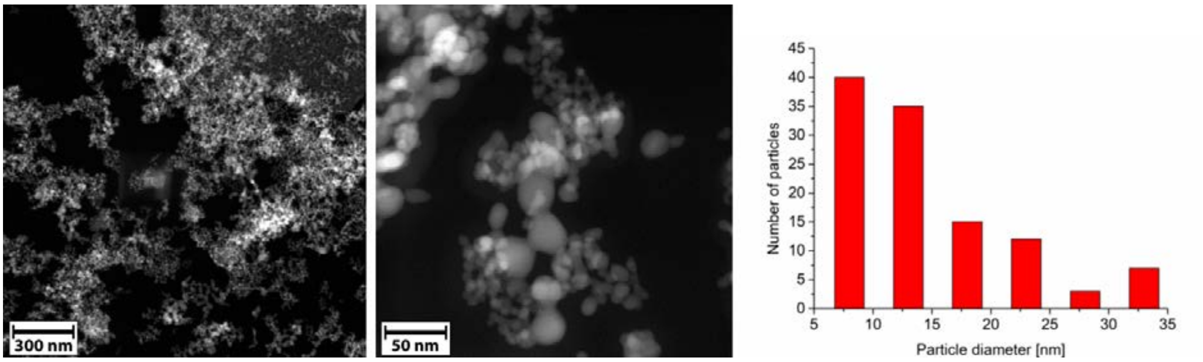

Figure S4a: HAADF-STEM images and particle size histogram of 1.0 wt % PrF_3-NPs in [BMIm][BF_4] from Pr(amd)$_3$.

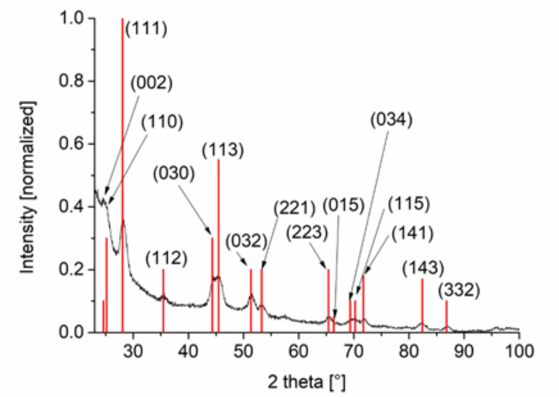

Figure S4b: PXRD (PrF_3 reference peaks in red from COD 1010984, hexagonal with space group P6$_3$/mcm) of 1.0 wt % PrF_3-NPs in [BMIm][BF_4] from Pr(amd)$_3$.

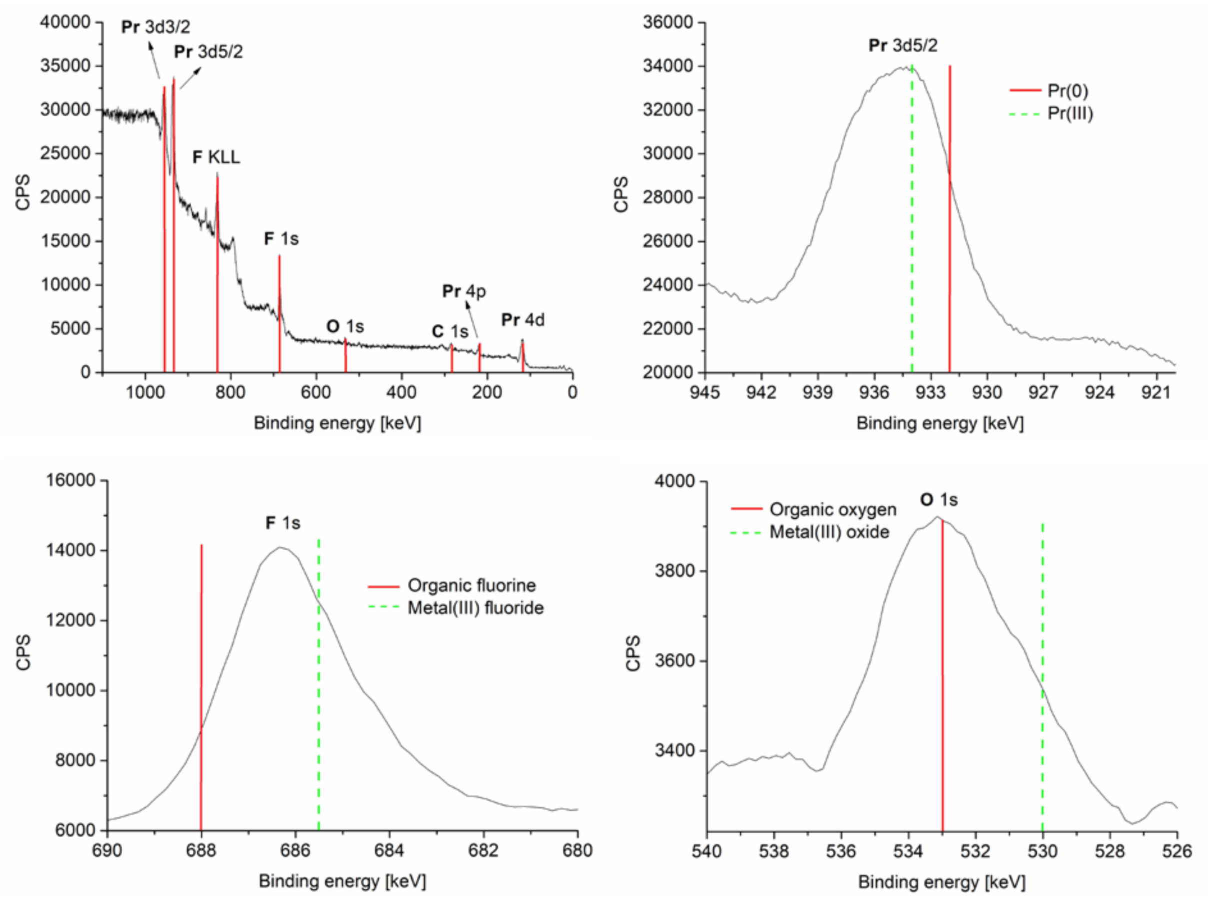

Figure S4c: Overview and HR-XPS of 1.0 wt % PrF_3-NPs in [BMIm][BF_4] from Pr(amd)$_3$. The red and green bars are a guide to the eye on the binding-energy axis.

EuF_3-NPs in [BMIm][BF_4]

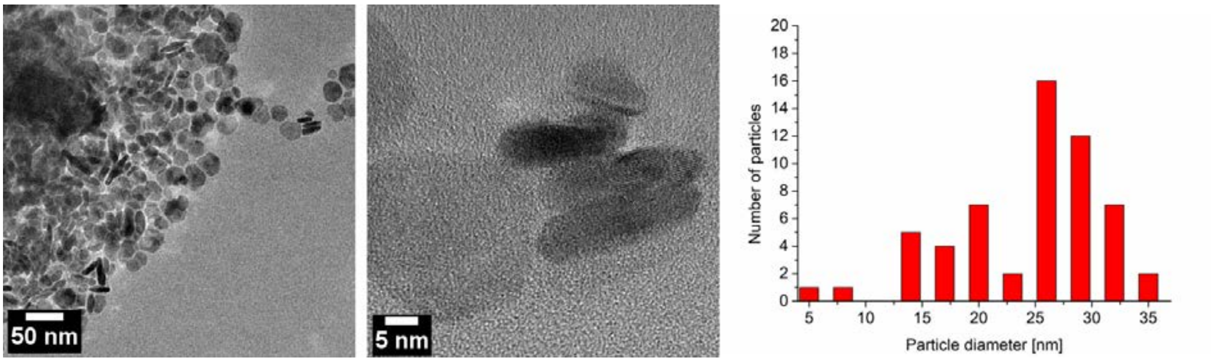

Figure S5a: TEM images and particle size histogram of 1.0 wt % EuF_3-NPs in [BMIm][BF_4] from Eu(dpm)$_3$.

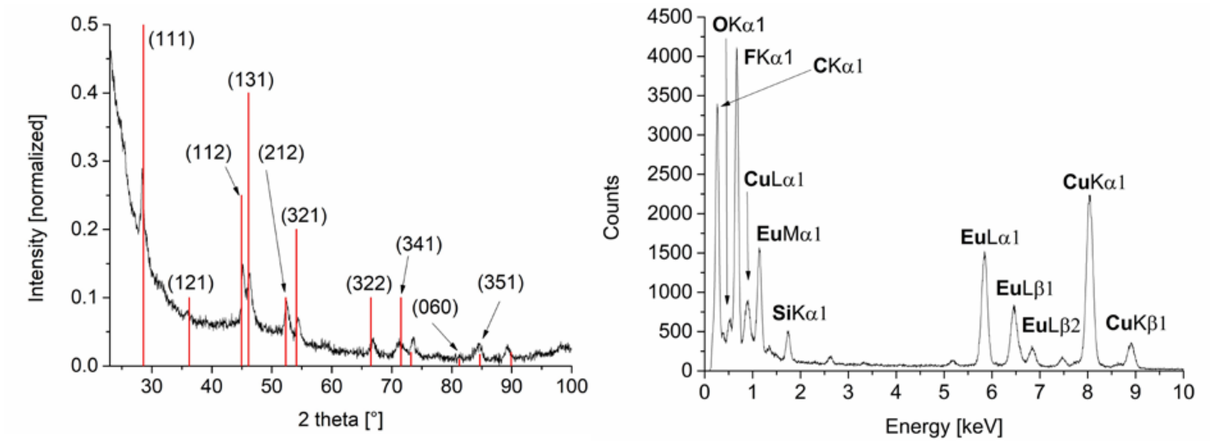

Figure S5b: PXRD (EuF_3 reference peaks in red from COD 1532444, orthorhombic with space group Pnma) and EDX of 1.0 wt % EuF_3-NPs in [BMIm][BF_4] from Eu(dpm)$_3$.

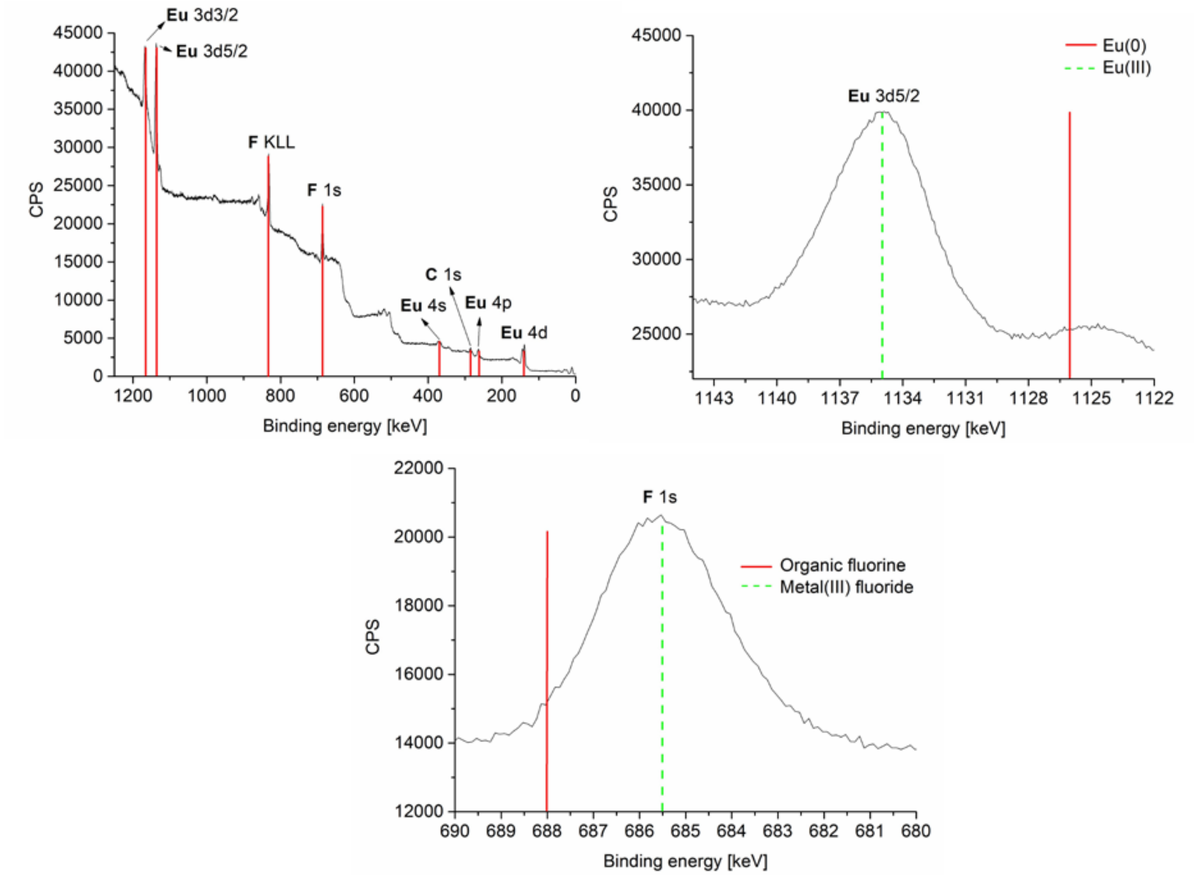

Figure S5c: Overview and HR-XPS of 1.0 wt % EuF_3-NPs in [BMIm][BF_4] from Eu(dpm)$_3$. The red and green bars are a guide to the eye on the binding-energy axis.

GdF_3-NPs in [BMIm][BF_4]

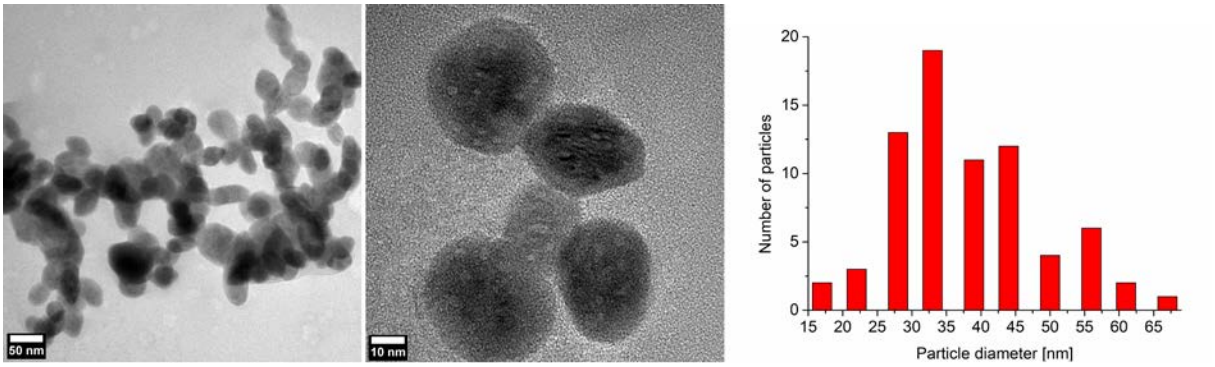

Figure S6a: TEM images and particle size histogram of 1.0 wt % GdF_3-NPs in [BMIm][BF_4] from Gd(amd)$_3$.

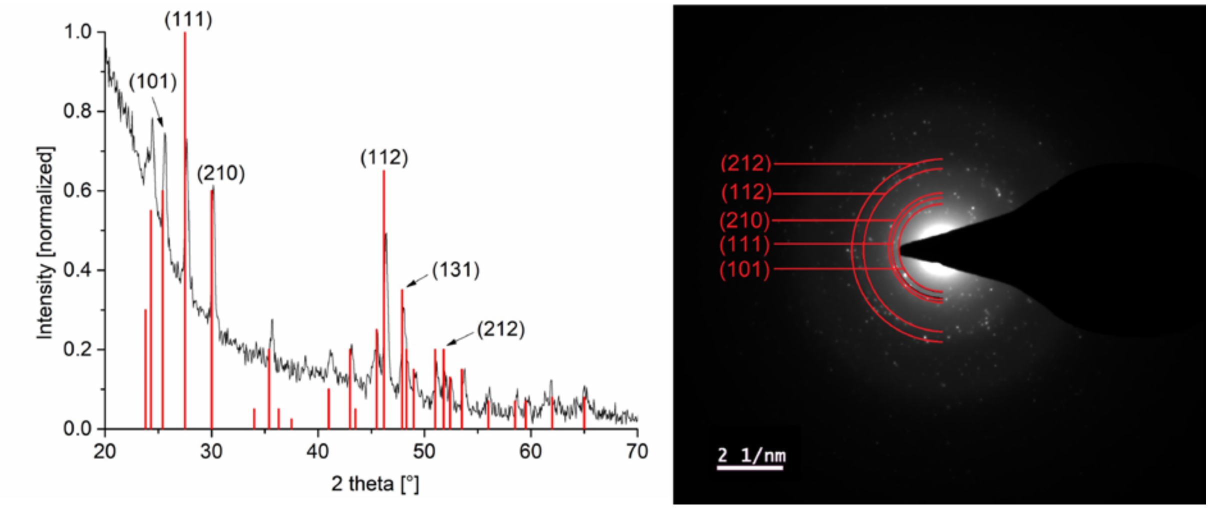

Figure S6b: PXRD and SAED (GdF_3 reference peaks in red from ICDD 0120788, orthorhombic with space group *Pnma*) of 1.0 wt % GdF_3-NPs in [BMIm][BF_4] from Gd(amd)$_3$.

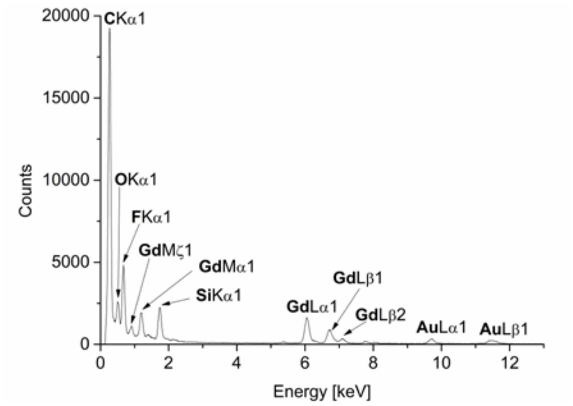

Figure S6c: EDX of 1.0 wt % GdF_3-NPs in [BMIm][BF_4] from $Gd(amd)_3$.

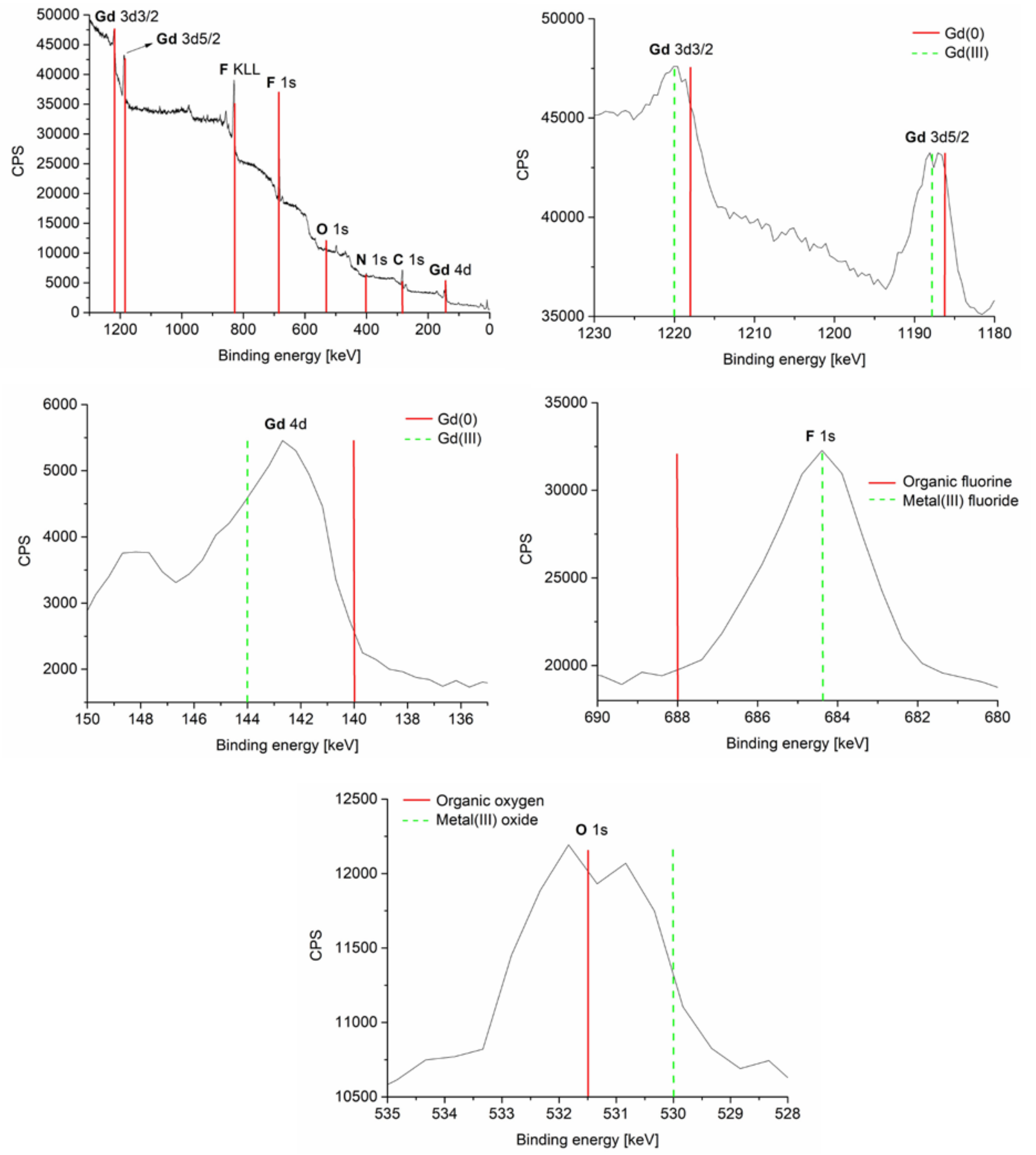

Figure S6d: Overview and HR-XPS of 1.0 wt % GdF_3-NPs in [BMIm][BF_4] from $Gd(amd)_3$. The red and green bars are a guide to the eye on the binding-energy axis.

TEM images, particle size histogram, SAED, EDX and XPS of Er-NPs in [BMIm][NTf$_2$]

Er-NPs in [BMIm][NTf$_2$]

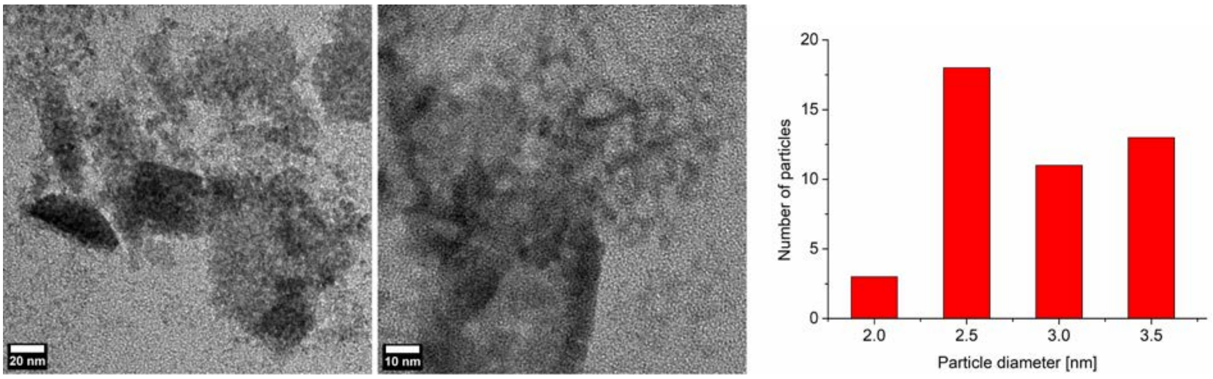

Figure S7a: TEM images and particle size histogram of 1.0 wt % Er-NPs in [BMIm][NTf$_2$] from Er(amd)$_3$.

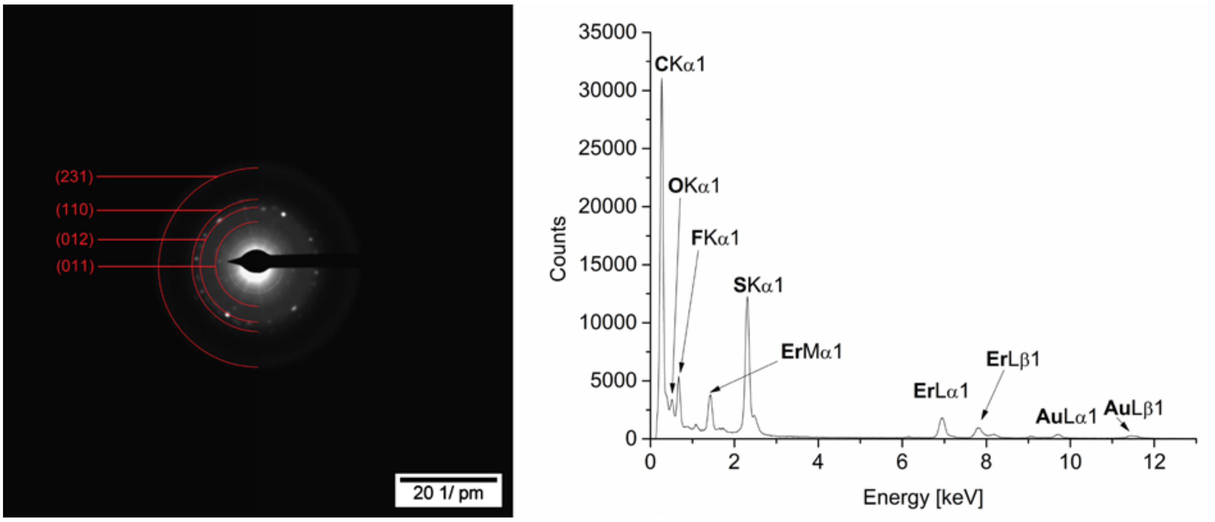

Figure S7b: SAED (Er reference peaks in red from COD 9008496, hexagonal with space group*P*6$_3$/*mcm*) and EDX of 1.0 wt % Er-NPs in [BMIm][NTf$_2$] from Er(amd)$_3$.

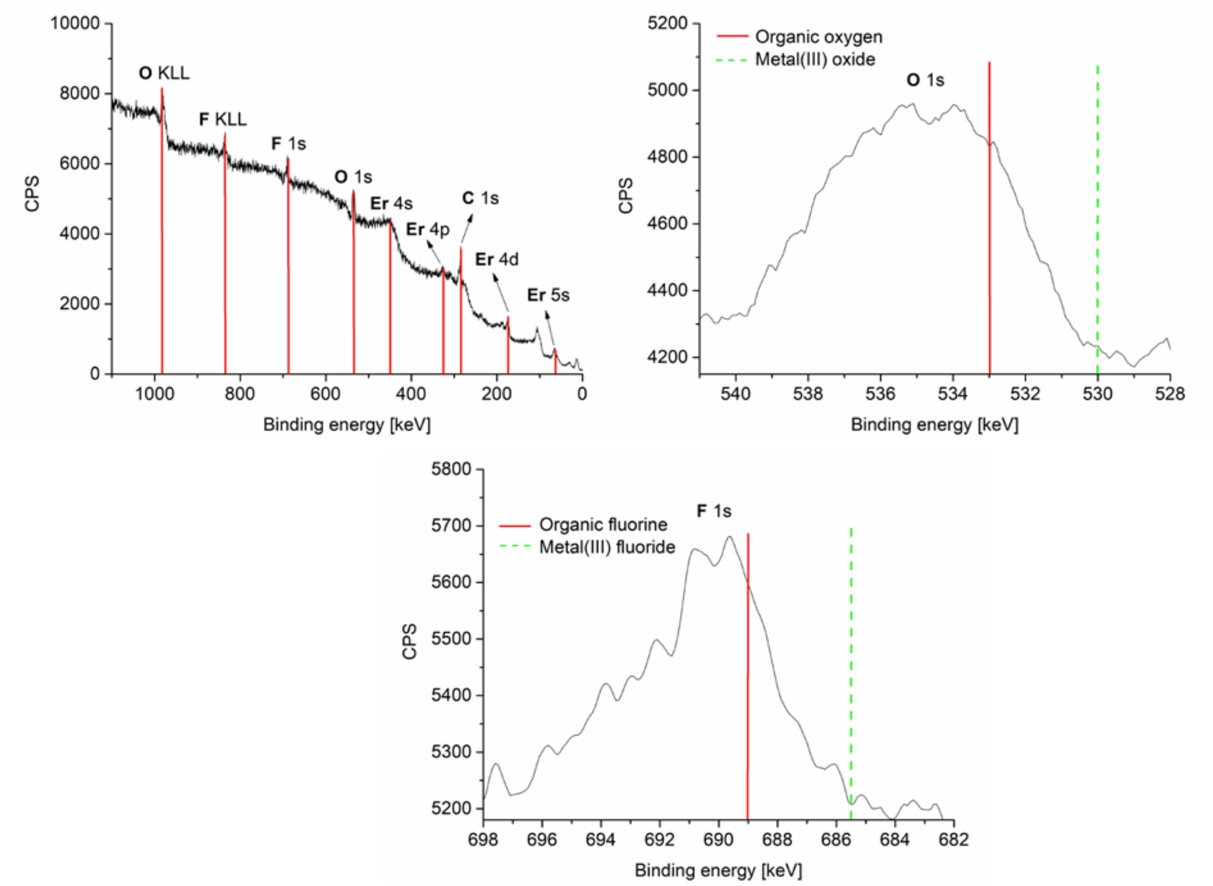

Figure S7c: Overview and HR-XPS of 1.0 wt % Er-NPs in [BMIm][NTf_2] from Er(amd)$_3$. The red and green bars are a guide to the eye on the binding-energy axis.

TEM images, particle size histogram, SAED, EDX and XPS of RE-NPs in PC

Pr-NPs in PC

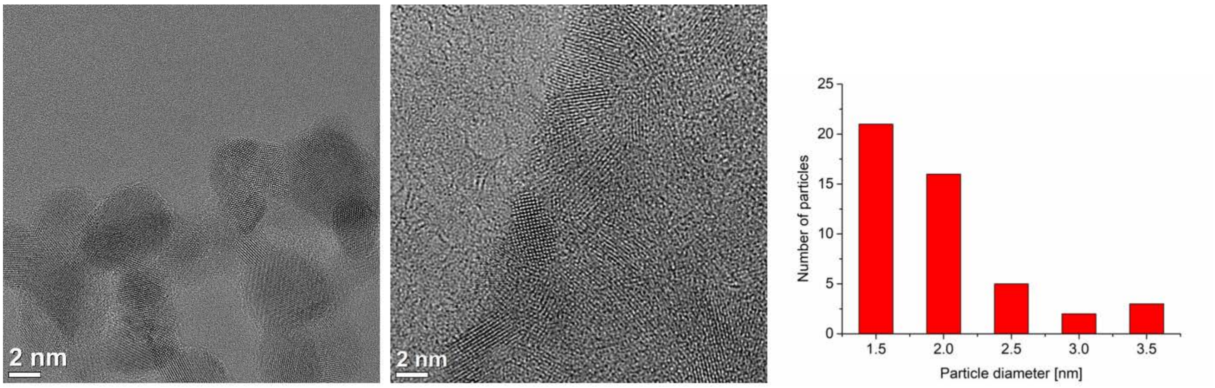

Figure S8a: TEM images and particle size histogram of 1.0 wt % Pr-NPs in PC from $Pr(amd)_3$.

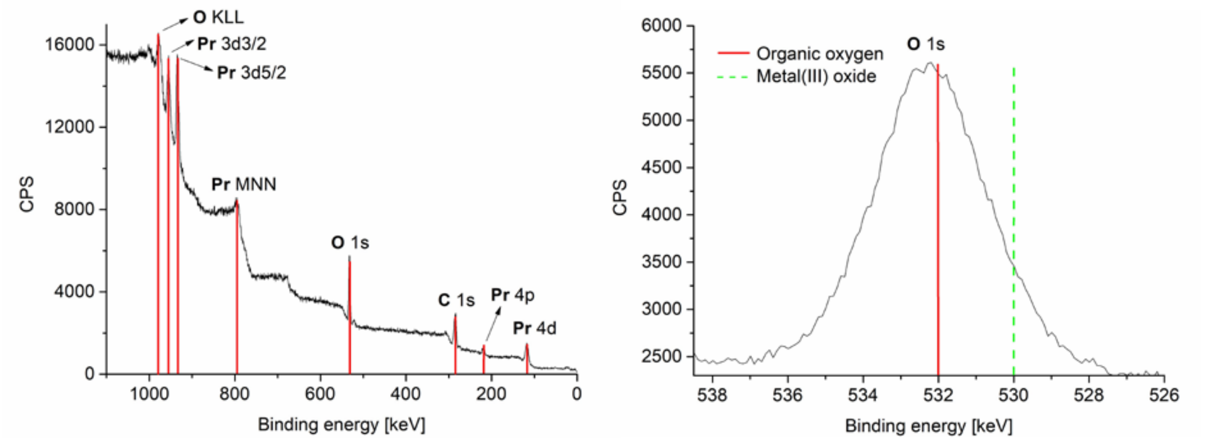

Figure S8b: Overview and HR-XPS of 1.0 wt % Pr-NPs in PC from $Pr(amd)_3$. The red and green bars are a guide to the eye on the binding-energy axis.

Er-NPs in PC

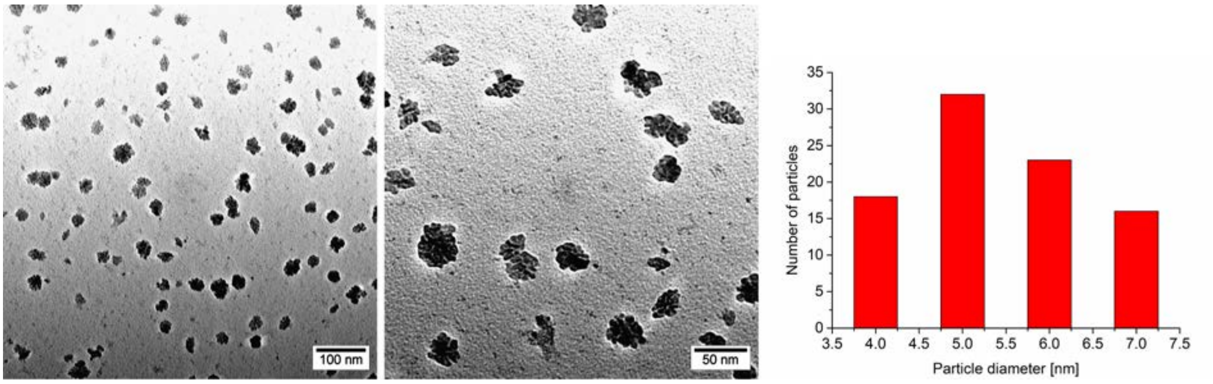

Figure S9a: TEM images and particle size histogram of 1.0 wt % Er-NPs in PC from Er(amd)$_3$.

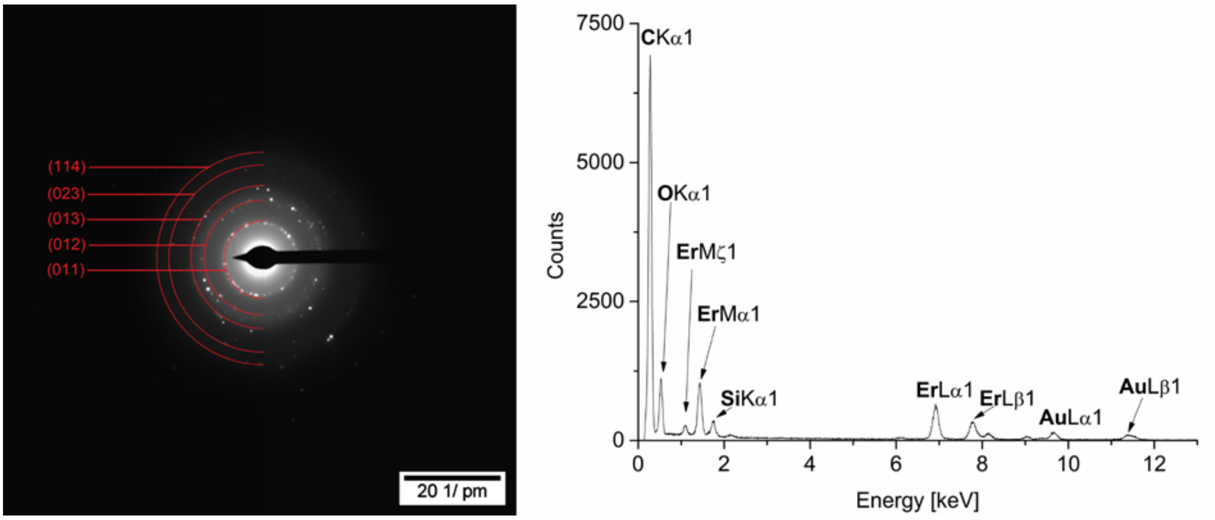

Figure S9b: SAED (Er reference peaks in red from COD 9008496, hexagonal with space group $P6_3/mcm$) and EDX of 1.0 wt % Er-NPs in PC from Er(amd)$_3$.

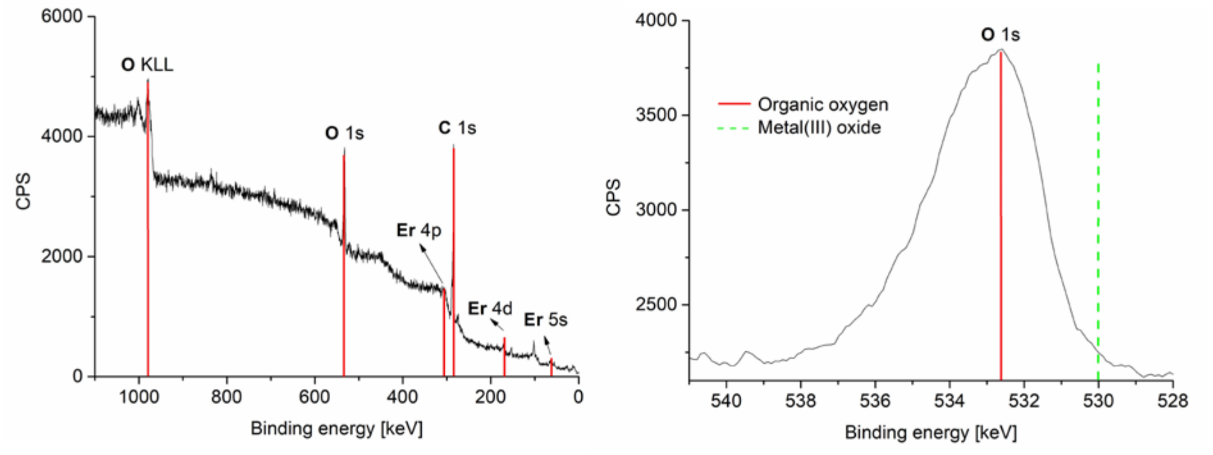

Figure S9c: Overview and HR-XPS of 1.0 wt % Er-NPs in PC from $Er(amd)_3$. The red and green bars are a guide to the eye on the binding-energy axis.
